# XAB2 functions in mitotic cell cycle progression via transcriptional regulation of CENPE

**DOI:** 10.1038/cddis.2016.313

**Published:** 2016-10-13

**Authors:** Shuai Hou, Na Li, Qian Zhang, Hui Li, Xinyue Wei, Tian Hao, Yue Li, Sikandar Azam, Caigang Liu, Wei Cheng, Bilian Jin, Quentin Liu, Man Li, Haixin Lei

**Affiliations:** 1 Institute of Cancer Stem Cell, Cancer Center, Dalian Medical University, Dalian, China; 2 Department of Oncology, Second Affiliated Hospital, Institute of Cancer Stem Cell, Dalian Medical University, Dalian, China; 3 Breast Disease and Reconstruction Center, Breast Cancer Key Lab of Dalian, Second Affiliated Hospital, Dalian Medical University, Dalian, China

## Abstract

Xeroderma pigmentosum group A (XPA)-binding protein 2 (XAB2) is a multi-functional protein that plays critical role in processes including transcription, transcription-coupled DNA repair, pre-mRNA splicing, homologous recombination and mRNA export. Microarray analysis on gene expression in XAB2 knockdown cells reveals that many genes with significant change in expression function in mitotic cell cycle regulation. Fluorescence-activated cell scanner analysis confirmed XAB2 depletion led to cell arrest in G2/M phase, mostly at prophase or prometaphase. Live cell imaging further disclosed that XAB2 knockdown induced severe mitotic defects including chromosome misalignment and defects in segregation, leading to mitotic arrest, mitotic catastrophe and subsequent cell death. Among top genes down-regulated by XAB2 depletion is mitotic motor protein centrosome-associated protein E (CENPE). Knockdown CENPE showed similar phenotypes to loss of XAB2, but CENPE knockdown followed by XAB2 depletion did not further enhance cell cycle arrest. Luciferase assay on CENPE promoter showed that overexpression of XAB2 increased luciferase activity, whereas XAB2 depletion resulted in striking reduction of luciferase activity. Further mapping revealed a region in CENPE promoter that is required for the transcriptional regulation by XAB2. Moreover, ChIP assay showed that XAB2 interacted with CENPE promoter. Together, these results support a novel function of XAB2 in mitotic cell cycle regulation, which is partially mediated by transcription regulation on CENPE.

Xeroderma pigmentosum group A (XPA)-binding protein 2 (XAB2) is a highly conserved gene, which was originally identified in human as a protein interacting with XPA using yeast two-hybrid system.^1^ The human XAB2 gene is located on chromosome 19p13.2, encoding a protein of 855 amino acids with molecular weight of 100 kDa. The XAB2 protein contains 15 tetratricopeptide repeat motifs involved in protein–protein interactions and the assembly of multiprotein complexes. It has many orthologues, such as SYF1 in *Saccharomyces cerevisiae*,^2, 3^ ATH55 in rat^4^ and Fandango in Drosophila.^5^

It was reported that XAB2 could interact with Cockayne syndrome group A and B (CSA and CSB) proteins as well as RNA polymerase II, and downregulation of XAB2 using anti-XAB2 antibody or siRNA both specifically inhibited normal RNA synthesis and the recovery of RNA synthesis after UV irradiation, indicating that XAB2 is involved in transcription and transcription-coupled DNA repair.^1, 6^ Further studies showed that XAB2 might bind to hyperphosphorylated form of RNA polymerase II in a UV- and CSA/CSB-dependent manner^7^ and contribute to transcription elongation.^6^ Furthermore, XAB2 is implicated as a component of Prp19/XAB2 complex^6^ (hAquarius, XAB2, Prp19, CCDC16, hISY1 and PPIE) or Prp19/CDC5L-related complex^8^ required for pre-mRNA splicing. During splicing, Prp19 complex is recruited to the spliceosome and crucial for spliceosome activation by stabilizing U5 and U6 with the spliceosome after U4 dissociation.^9, 10^ In Hela cells, Prp19/XAB2 complex was reported to associate with RNA rather than DNA, and knockdown of XAB2 would inhibit Bcl-x pre-mRNA splicing.^6^ Similarly, SYF1, homolog of XAB2 in yeast, is also identified as a factor involved in pre-mRNA splicing,^2, 3^ and a novel transcription elongation factor by linking Prp19 complex with RNA polymerase II through its C terminal domain.^11^

XAB2 is also essential for early mouse embryogenesis, because knockdown of XAB2 in mice has been shown to lead to embryonic lethality.^12^ It was previously demonstrated that XAB2 could interact with retinoic acid receptor A and histone deacetylase 3, and XAB2 deficiency increased all-trans retinoic acid (ATRA)-induced cellular differentiation in ATRA-sensitive and -resistant cancer cells. These results suggest that XAB2 may display an inhibitory effect in ATRA-induced cellular differentiation.^13^ Furthermore, XAB2 may play a role in tumorigenesis. XAB2 expression is significantly downregulated in primary gastric cancer,^14^ triple-negative breast cancer^15^ and a long-term survivor of an atypical teratoid/rhabdoid tumour,^16^ while it is upregulated in ovarian cancer^17^ and sarcoma^18^ using Oncomine database (www.oncomine.org). The XAB2 tagSNPs (rs794078 and rs4134816) are dramatically correlated with the risk of non-small cell lung cancer in Chinese population.^19^ In addition, XAB2 levels are strikingly reduced in aged hematopoietic stem cells, suggesting a role in ageing.^20^ Recently, we showed that XAB2 presented in the ribonucleo-protein of a consensus element found in naturally intronless mRNAs that can promote mRNA export, and disruption of XAB2 led to nuclear retention of the intronless mRNAs, suggesting a role of XAB2 in naturally intronless mRNA export.^21^ Most recently, XAB2 has been reported to regulate the end resection step during homologous recombination repair of chromosomal double-strand breaks.^22^ Thus, XAB2 is an important multifunctional protein.

Despite the critical functions of XAB2 in gene expression, the downstream target genes and the subsequent physiological roles in human cells are still unknown. Here, we perform microarray analysis on Hela cells transfected with XAB2 shRNA, revealing that XAB2 modulates the expression of a significant number of genes, most highly involved in cell cycle and mitotic progression. We further show that XAB2 knockdown causes chromosome misalignment and missegregation, abnormal nuclear structure, spindle pole, and microtubule organization, leading to mitotic arrest, mitotic catastrophe and subsequent cell death. Mechanistic analysis reveals that the phenotype observed in XAB2 depletion cells is mediated by a kinesin-like motor protein called centrosome-associated protein E (CENPE), the transcription of which is regulated by XAB2. Taken together, our data indicate that XAB2 regulates mitotic progression by transcription control of CENPE.

## Results

### Microarray analysis reveals that XAB2 knockdown results in aberrant expression of many genes involved in mitotic cell cycle

**Figure 1:**
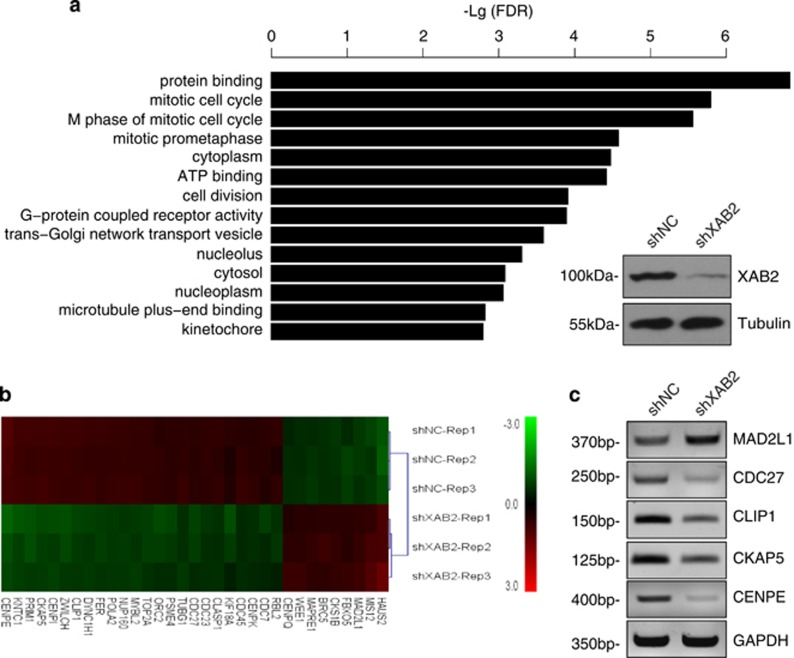
Microarray analysis reveals genes involved in cell cycle and mitotic progression as major genes with expression changes after XAB2 knockdown. (**a**) Gene Ontology analysis showing gene functions with significant changes in expression after XAB2 knockdown. Right-bottom panel: western blot showing XAB2 was efficiently depleted after cells were infected with lentivirus containing XAB2 shRNA. (**b**) Differential expression of genes (>2-fold, *P*<0.05) related to cell cycle and mitotic progression after XAB2 knockdown. (**c**) RT-PCR analysis validated gene expression levels after XAB2 knockdown

Previous studies indicated that XAB2 is a multifunctional protein that plays critical role in transcription,^6^ splicing^6^ and mRNA export.^21^ To identify target genes regulated by XAB2, gene expression of XAB2 depleted HeLa cells was analysed by microarray. This analysis revealed that a total of 690 genes showed significant change in expression (>2-fold, *P*<0.05). Among them, 216 genes were up-regulated and 474 genes were down-regulated (Supplementary Table 1). The knockdown efficiency of XAB2 was confirmed by western blot (Figure 1a) at protein level as well as by microarray at mRNA level (Supplementary Table 1, down-regulated 3.43-fold). To verify microarray data, RT-PCR was used to check the expression level of several genes including MAPK4, SERPINB5, EPGN and PRG4. Consistent with the microarray results, reduced expression of these four genes was confirmed by RT-PCR (Supplementary Figure S1).

Next, Gene Ontology analysis was performed to analyse the biological functions of the 690 genes with altered expression. As shown in Figure 1a, several top functions affected were related to cell cycle regulation, including mitotic cell cycle, M phase of mitotic cell cycle, mitotic prometaphase and cell division. Accordingly, a set of genes involved in the regulation of cell cycle were deregulated (Figure 1b), including CENPE, CKAP5, CLIP1, CDC27 and Mad2L1, the expression of which were validated by RT-PCR (Figure 1c). Together, these data suggested a critical role of XAB2 in mitosis and cell cycle regulation.

### XAB2 knockdown leads to mitotic arrest, mitotic catastrophe and cell death

**Figure 2:**
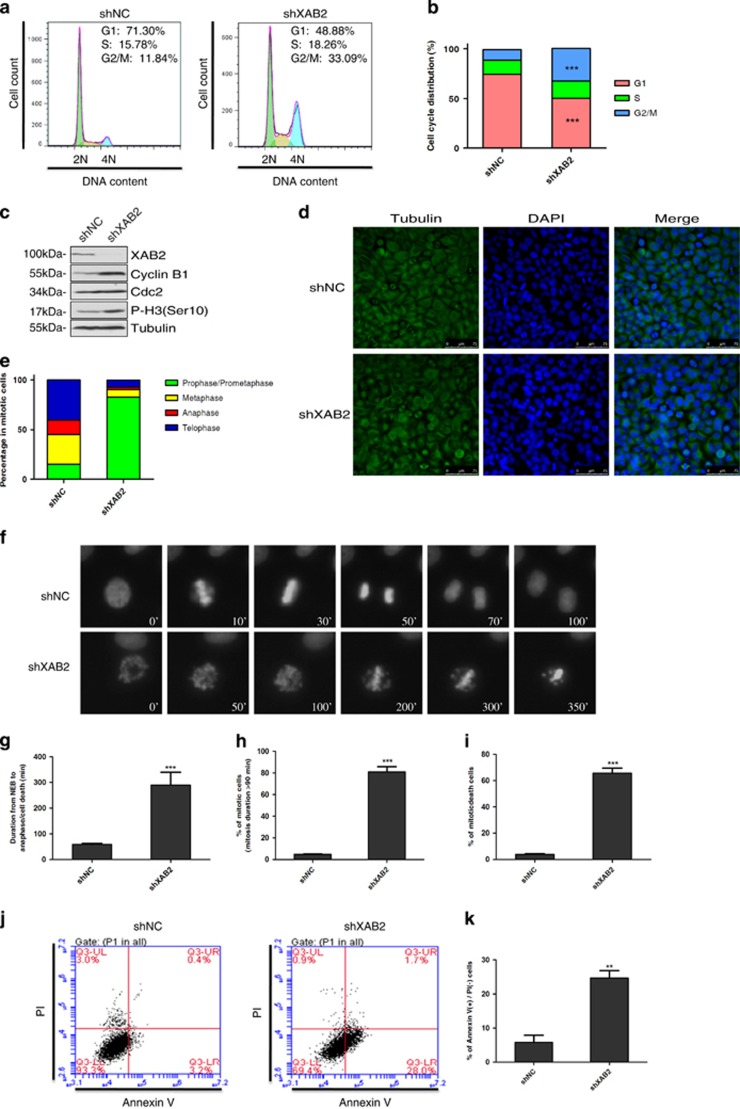
XAB2 deficiency causes mitotic arrest, mitotic catastrophe and cell death. (**a**) Depletion of XAB2 results in accumulation of G2/M cells by FACS analysis. Hela cells were transfected with control shRNA (shNC) or XAB2 shRNA (shXAB2) and then stained with PI for analysis of cell cycle distribution. (**b**) Quantitation of cells at different cell cycle phases after XAB2 depletion (*n*=3); ***P≤0.001. (**c**) Western blot reveals upregulation of CyclinB1 and Phospho-HistoneH3 (Ser10) in XAB2 depleted cells. (**d**) XAB2 knockdown induces significant increase of cells in prophase and prometaphase. Hela cells were transfected with control shRNA (shNC) or XAB2 shRNA (shXAB2) and then immunostained with *α*-tubulin antibody and DAPI. (**e**) Quantitation of mitotic cells at different phases after XAB2 depletion. More than 100 randomly selected mitotic cells were counted (*n*=3). (**f**) Live cell imaging showing XAB2 knockdown resulted in mitotic arrest and cell death. (**g**) Quantitation of average mitosis duration of GFP-H2B Hela cells with control or XAB2 knockdown (*n*=30 cells); ***P≤0.001 (NEB: nuclear envelop breakdown). (**h**) Quantitation of cells in mitotic arrest or mitotic delay (mitosis duration >90 min) as observed in live cell imaging. The mitosis duration is defined as the time lapse from nuclear envelop breakdown to anaphase onset or cell death; ***P≤0.001. (**i**) Quantitation of mitotic death cells after XAB2 depletion. More than 50 randomly selected mitotic cells were counted (*n*=3); ***P≤0.001. (**j**) XAB2 knockdown causes apoptosis by FACS analysis using annexin V and PI staining. (**k**) Quantitation of annexin V-positive cells after XAB2 depletion (*n*=3); **P≤0.01

To further investigate whether XAB2 regulates cell cycle and mitotic progression, fluorescence-activated cell scanner (FACS) analysis was performed on Hela cells transfected with XAB2 shRNA. Strikingly, XAB2 knockdown resulted in a significant increase of cells arrested in G2/M phase (32.8% in XAB2 shRNA transfected cells compared to 10.6% in control, *P*<0.001, Figures 2a and b). In order to rule out off-target effects of XAB2 shRNA, another shRNA and two siRNAs were tested for their effects on cell cycle. Similarly, knockdown XAB2 using different shRNA or siRNA resulted in significant cell cycle arrest at G2/M phase in both Hela (Supplementary Figure S2A) and 293T cells (Supplementary Figure S2B), indicating cell cycle arrest is a major defect after XAB2 depletion. Consistently, the expression of mitotic markers Cyclin B1 and Phospho-HistoneH3 (Ser10) was apparently increased in XAB2 knockdown cells by western blot (Figure 2c) and FACS analysis (Supplementary Figure S2C).

Next, XAB2 knockdown cells and control cells were stained with antibody against tubulin and DAPI, significant mitotic cells arrested at prophase and prometaphase were observed in cells with XAB2 depletion (82.8 versus 15.1% in control, *P*<0.001, Figures 2d and e), further supporting of severe defects in mitotic cell cycle progression.

To better investigate the function of XAB2 on mitotic progression, we depleted XAB2 in HeLa cells stably expressing GFP-H2B and tracked mitosis using live cell imaging. As shown in Figure 2f, two daughter nuclei were separated by 50 min and mitosis was completed by 70 min in control cells. In contrast, nuclei separation was not observed even by 300 min in XAB2 depleted cells. Statistic analysis further revealed that the average mitosis duration time from nuclear envelope breakdown to anaphase was prolonged from ~60 min in control cells to 290 min in cells with XAB2 depletion (Figure 2g, *P*<0.001). The percentage of cells showing mitotic arrest or mitotic delay (mitosis duration >90 min) was raised dramatically from 4.9 to 81.2% (Figure 2h, *P*<0.001). Furthermore, cells undergoing mitotic cell death were significantly increased after XAB2 knockdown from 3.9 to 65.8% (Figure 2i, *P*<0.001). To further test whether XAB2 knockdown induced cell death after mitotic catastrophe, FACS analysis was performed after annexin V and propidium iodide (PI) staining. As shown in Figures 2j and k, the percentage of annexin V-positive cells was obviously increased in XAB2 knockdown cells from 5.8 to 24.7%, suggesting significant cell death after XAB2 depletion. Taken together, these data indicated that XAB2 deficiency resulted in mitotic arrest, mitotic catastrophe and cell death.

### XAB2 knockdown causes chromosome alignment and segregation defects

**Figure 3:**
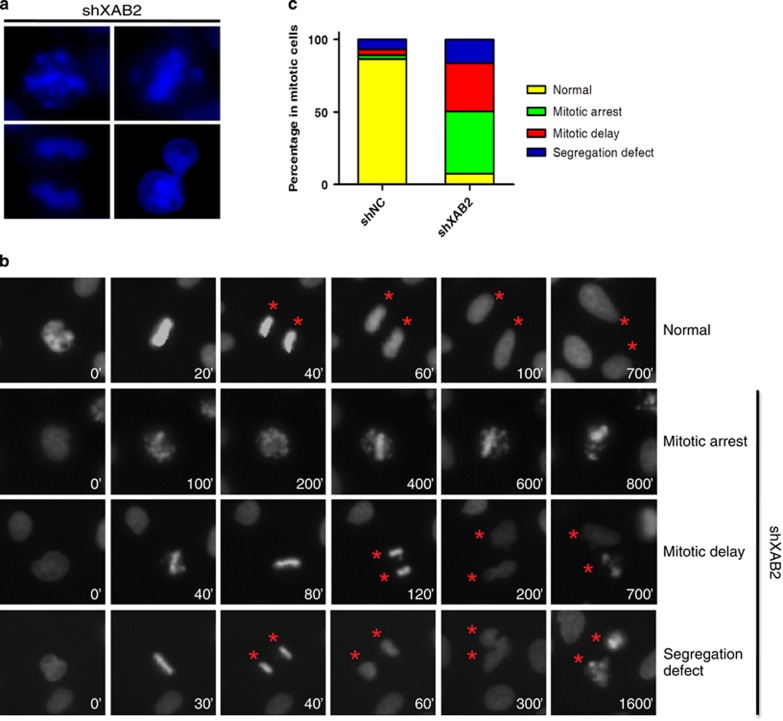
XAB2 knockdown leads to chromosome misalignment and missegregation. (**a**) Misaligned/missegregated chromosomes and internuclear bridges observed in XAB2-depleted cells. (**b**) Live cell imaging showing XAB2 knockdown resulted in mitotic arrest, mitotic delay and segregation defect. (**c**) Quantitation of mitotic cells after XAB2 depletion showed significant increase in mitotic arrest, mitotic delay and segregation defect

Because mitosis requires a tight control of chromosome movement, we next performed DAPI staining to determine whether XAB2 affects chromosome alignment and segregation. This analysis revealed severe chromosome misalignment at equatorial plate and internuclear DNA bridges (Figure 3a).

Consistent with previous observation, most of the mitotic cells showed mitotic arrest, mitotic delay or segregation defect. Specifically, mitotic arrest in prophase or prometaphase with defects in chromosome alignment was observed in 43.1% of mitotic cells after XAB2 depletion compared to 2.6% in control, which did not divide and eventually led to cell death (Figures 3b and c). Mitotic delay was observed in 33.2% of mitotic cells after XAB2 knockdown versus 4.2% in control, these cells were still able to divide after a mitotic delay with chromosome misalignment, but also with chromosome segregation defect resulting in the formation of internuclear DNA bridges (Figures 3b and c). Whereas segregation defect without chromosome misalignment was also observed in 16.3% of mitotic cells as a comparison to 6.9% in control (Figures 3b and c). Both cells of mitotic delay or segregation defect showed abnormal nuclear structure and died in cell cycle progression (Figure 3b). These data indicated severe defects in chromosome alignment and segregation after XAB2 knockdown.

### XAB2 deficiency induces abnormal spindle pole, nuclear structure and microtubule organization and DNA breaks

**Figure 4:**
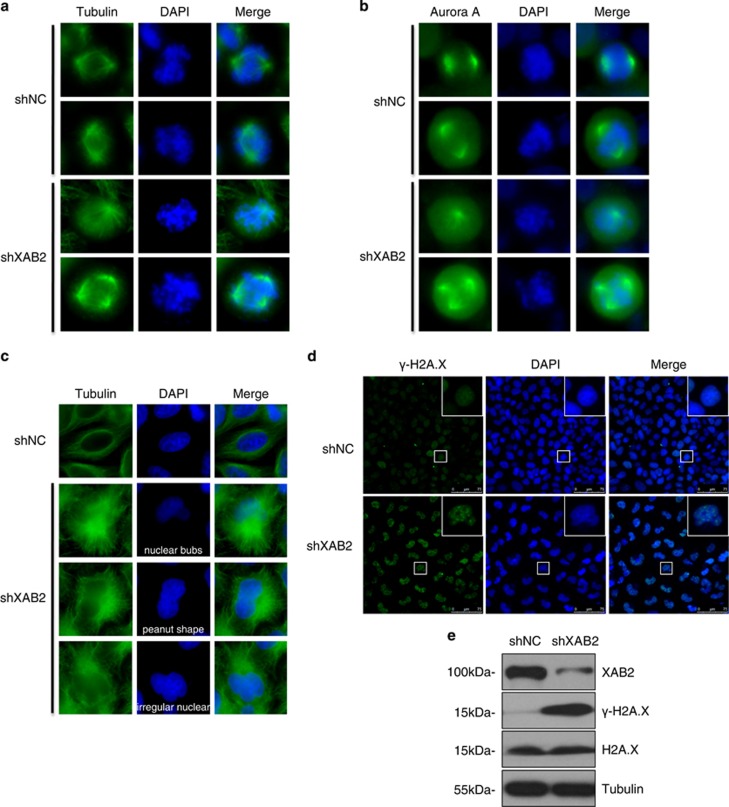
XAB2 knockdown results in aberrant spindle pole, disrupted microtubule organization and increased DNA damage. (**a**, **b**) Depletion of XAB2 leads to single or multiple spindle poles in prophase or prometaphase cells. Hela cells were transfected with control shRNA (shNC) or XAB2 shRNA (shXAB2) and then immunostained with *α*-tubulin antibody (**a**) or Aurora A antibody (**b**) and DAPI. (**c**) Disrupted microtubule organization and nuclear structure in interphase cells after XAB2 knockdown. Hela cells were immunostained with *α*-tubulin antibody (green) and DAPI (blue). (**d**) XAB2 knockdown results in increased DNA breaks as showed by immunostaining using *γ*-H2A.X antibody (green). (**e**) Western blot reveals upregulation of *γ*-H2A.X in XAB2 knockdown cells

We next sought to further examine how mitosis is disturbed in XAB2 knockdown cells. First, spindle pole was checked in XAB2 depleted cells since correct spindle formation is required for chromosome alignment and segregation. Strikingly, single or multiple spindle poles was observed in ~30 and ~8% of prophase/prometaphase cells after XAB2 knockdown using either tubulin or Aurora A staining (Figures 4a and b). Second, abnormal nuclear structures including nuclear bubs, peanut shape or irregular nucleus were observed in most of interphase cells after XAB2 depletion (Figure 4c). Meanwhile, microtubule organization was also severely disrupted. Clustered irregular assembly of microtubules was observed at one side of nucleus as compared to an even distribution in control cells (Figure 4c). Third, DNA damages in XAB2 knockdown cells were examined since nuclear bubs are generally associated with DNA breaks.^23, 24^ As shown in Figures 4d and e, the expression of DNA damage marker Phospho-Histone H2A.X (*γ*-H2A.X) was significantly increased in XAB2 knockdown cells by both immunofluorescence staining and western blot analysis. Together, these data suggested that XAB2 was critical for microtubule and spindle organization as well as genome stability.

### Defects in mitotic progression in XAB2 depletion cells are mediated by CENPE

**Figure 5:**
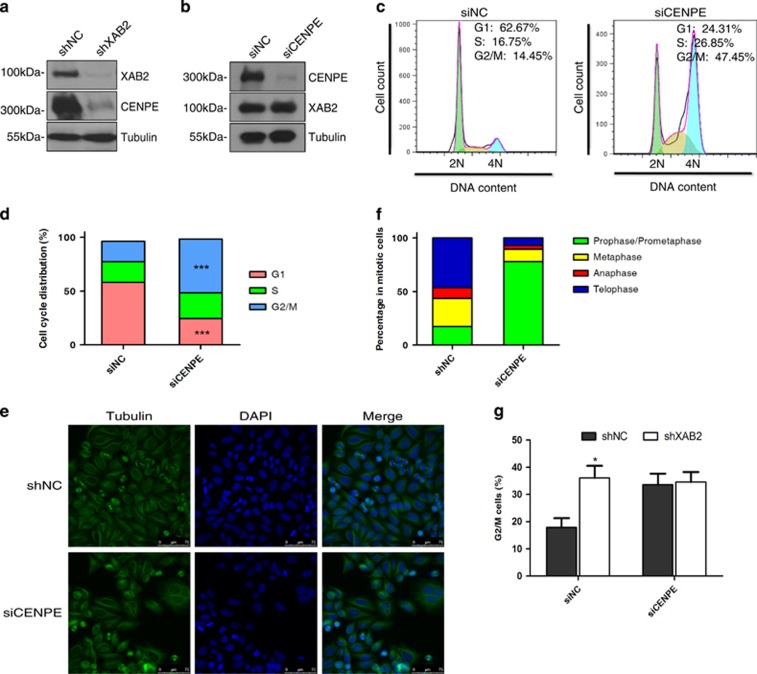
XAB2 regulates mitotic progression via CENPE. (**a**) Western blot showing decreased expression of CENPE protein in XAB2 knockdown cells. (**b**) Western blot showing knockdown of CENPE did not change the level of XAB2 protein. (**c**) CENPE knockdown leads to G2/M arrest as revealed by FACS analysis. Hela cells were transfected with control siRNA (siNC) or CENPE siRNA (siCENPE) and then stained with PI to analyse cell cycle distribution. (**d**) Quantitation of cells at different cell cycle phases after CENPE depletion (*n*=3); ***P≤0.001. (**e**) Immunofluorescence staining shows significant increase of cells in prophase and prometaphase after CENPE knockdown. Hela cells were transfected with control siRNA (siNC) or CENPE siRNA (siCENPE) and then immunostained with *α*-tubulin antibody and DAPI. (**f**) Quantitation of mitotic cells at different phases after CENPE depletion. More than 50 randomly selected mitotic cells were counted (*n*=3). (**g**) CENPE knockdown followed by XAB2 shRNA treatment does not further increase G2/M arrest induced by CENPE depletion. (*n* = 3); *P⩽0.05

We next set out to explore the mechanism on how XAB2 regulates mitosis. We noticed that CENPE, a key factor in mitosis, was among the top genes down-regulated in XAB2 knockdown microarray results. Therefore, we tested the hypothesis that XAB2 regulates mitosis via CENPE. First, expression level of CENPE in XAB2 depleted cells was verified by RT-PCR and western blot analysis, the results indicated that XAB2 depletion led to a dramatic loss of CENPE in both mRNA and protein level (Figures 1c and 5a, Supplementary Figure S3). Whereas knockdown CENPE showed no effect on XAB2 protein expression, suggesting XAB2 functions upstream of CENPE (Figure 5b). Next, CENPE was depleted in HeLa cells using CENPE specific siRNA, the depletion led to 50.0% of cells arrested at G2/M phase compared to 19.0% in control (Figures 5c and d, *P*<0.001). Meanwhile, CENPE depletion also induced chromosome misalignment and prophase/prometaphase arrest (78.1% in knockdown cells versus 17.2% in control), similar to the phenotype observed in XAB2 deficiency cells (Figures 5e and f). To further confirm whether XAB2 regulates mitosis via CENPE, HeLa cells were treated with CENPE siRNA followed by depletion of XAB2. As shown in Figure 5g, CENPE knockdown alone dramatically arrested cells in G2/M phase, whereas CENPE knockdown followed by XAB2 shRNA treatment did not further enhance the G2/M arrest caused by CENPE, illustrating the important role of CENPE in the mitotic progression control induced by XAB2 depletion. Together, these results suggested that XAB2 regulated mitosis via CENPE.

### XAB2 regulates CENPE expression at transcription level

**Figure 6:**
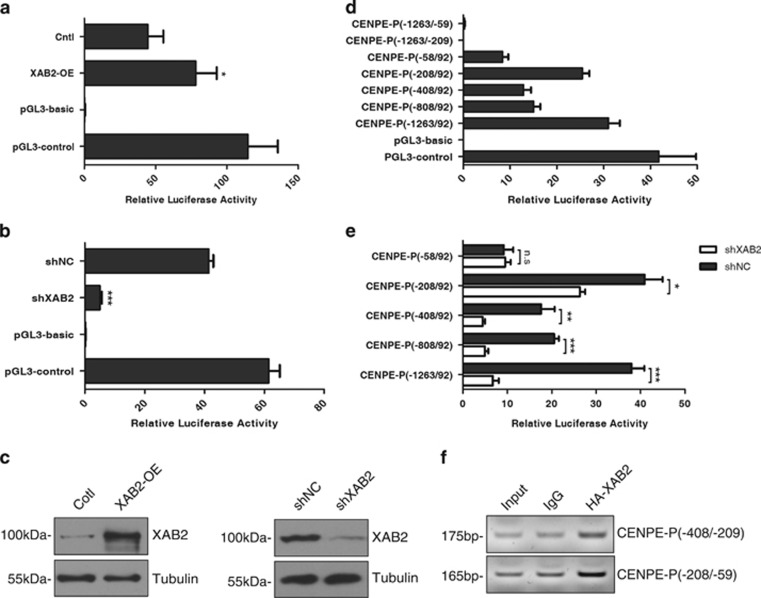
XAB2 regulates CENPE expression at transcriptional level. (**a**) Luciferase assay reveals that XAB2 overexpression increases CENPE promoter activity. Cntl: control, OE: over-expression (*n* = 3); *P⩽0.05. (**b**) Luciferase assay showing XAB2 knockdown decreases CENPE promoter activity. (*n* = 3); ***P⩽0.001. (**c**) Western blot shows the overexpression and knockdown efficiency of XAB2. (**d**) Luciferase assay of serial deletion constructs of CENPE promoter leads to the identification of core region of CENPE promoter. (**e**) Effect of XAB2 knockdown on deletion constructs of CENPE promoter by luciferase assay (*n* = 3); n.s., no significance, *P⩽0.05, **P⩽0.01, ***P⩽0.001. (**f**) ChIP assay shows XAB2 interacts with CENPE promoter

We next investigated how XAB2 might regulate CENPE expression. XAB2 was previously reported to interact with RNA polymerase II and play a role in transcription,^1, 6^ moreover, we observed that depletion of XAB2 led to down-regulation of CENPE at mRNA level in this study, suggesting XAB2 may transcriptionally regulate the expression of CENPE. To test this, we first constructed a luciferase reporter that spanned a 1355-bp region from −1263 to +92 of transcription starting site of CENPE. As shown in Figure 6a, this region could drive the transcription of luciferase, suggesting it did serve as CENPE promoter. Overexpression of XAB2 increased CENPE promoter activity by 1.8-fold (Figure 6a), whereas knockdown of CENPE resulted in a striking decrease of luciferase activity by 7.2-fold (Figure 6b). The overexpression and knockdown efficiency of XAB2 were confirmed by western blot analysis (Figure 6c).

To identify the minimal region in CENPE promoter required for XAB2 regulation, we generated a series of deletion constructs. Transfection of these constructs into HeLa cells indicated that the region −208/92 showed a luciferase activity close to the full length promoter (Figure 6d). Moreover, the region −1263/−209 had no promoter activity (Figure 6d), suggesting the core promoter spans about 300 bp from −208 to 92. Transfection of the deletion constructs followed by XAB2 knockdown revealed that XAB2 depletion resulted in striking decrease in luciferase activity for the full length promoter −1263/92, or deletion constructs −808/92 and −408/92, whereas further deletion −208/92 led to much less reduction and the decrease was abolished for −58/92 construct (Figure 6e), suggesting that the region between −408/−59 is critical for the transcriptional regulation of CENPE expression by XAB2. Chromatin immunoprecipitation (ChIP) of HA-tagged XAB2 protein also revealed significant enrichment of XAB2 at CENPE promoter (Figure 6f). Altogether, these data support that XAB2 transcriptionally regulates the expression of CENPE.

## Discussion

XAB2 is a component of Prp19 complex, it has been reported to function in transcription-coupled DNA repair, pre-mRNA splicing, mRNA export, transcription and homologous recombination. In this study, we reported a novel function of XAB2 as mitotic cell cycle regulator.

To gain deep insight into the impact of XAB2 deficiency, we performed microarray analysis on gene expression after XAB2 depletion in HeLa cells. Surprisingly, this analysis revealed that the expression of many genes functioning in cell cycle or mitosis regulation was changed significantly. We further showed that XAB2 deficiency induced chromosome misalignment and missegregation, abnormal nuclear structure, spindle pole, and microtubule organization, resulting in mitotic arrest, mitotic catastrophe and subsequent cell death, indicating a critical role of XAB2 in the regulation of mitotic cell cycle. Our results are consistent with phenotypes of XAB2 knockdown documented in the MITOCHECK database (www.mitocheck.org), including metaphase delay, metaphase alignment problems and cell deaths.^25^

In understanding how XAB2 affects mitotic cell cycle, CENPE turned out to be among the top genes with striking reduction in expression after knockdown of XAB2. CENPE is a plus end-directed kinetochore motor protein belonging to the kinesin-7 subfamily and has been extensively studied. It is first recruited at kinetochore during prometaphase depending on proteins such as BubR1,^26^ CENPF,^26^ NUF2,^27^ SEPT7,^28^ TRAMM^29^ and CTCF,^30^ and then degraded by APC/C and SCF at the end of mitosis.^31, 32^ CENPE is a very large protein about 312 kDa with multiple function domains, including an N-terminal ATP-dependent motor domain, a coiled-coil domain, a C-terminal kinetochore-binding domain and a C-terminal microtubule-binding domain.^26, 30^ Previous work has suggested that CENPE plays an important role in the chromosome congression during prometaphase,^33, 34^ the formation of stable attachment between spindle microtubules and kinetochores from prometaphase to anaphase,^35, 36^ and the microtubule plus-end elongation.^37^ Furthermore, CENPE is also essential for regulating SAC, probably by modulating BubR1 activities.^38, 39, 40, 41^ Downregulation of CENPE using various methods in different cell types and species consistently leads to chromosome misalignment and subsequent delayed mitotic progression.^33, 34, 38, 40, 42, 43, 44^ However, they show diverse cell fates, with some causing long-term mitotic arrest,^40, 42^ some resulting in chromosome missegregation after a mitotic delay,^38, 43, 44^ and some even leading to cell death by apoptosis.^33^

Thus we tested the possibility that XAB2 regulates mitotic cell cycle via CENPE. Indeed, XAB2 depletion led to dramatic decrease of CENPE at both mRNA and protein levels. Consistent with previous reports, CENPE depletion resulted in similar phenotypes as observed for XAB2 knockdown, including chromosome misalignment and mitotic arrest. Furthermore, CENPE knockdown followed by XAB2 shRNA treatment did not change the G2/M arrest caused by CENPE. These results suggested that XAB2 functions upstream of CENPE and may regulate mitotic cell cycle progression via CENPE.

We further showed that XAB2 binds to the promoter of CENPE and regulates its expression using ChIP and luciferase assay. Overexpression of XAB2 led to higher luciferase activity whereas XAB2 depletion resulted in striking decrease of luciferase activity. Previous report showed that XAB2 interacts with RNA polymerase II and plays a role in transcription, mostly by modulating transcription elongation.^6, 10^ Since XAB2 complex (hAquarius, XAB2, Prp19, CCDC16, hISY1 and PPIE) has been reported to bind to RNA but not DNA *in vitro* and XAB2 contains 15 tetratricopeptide repeat motifs involved in protein–protein interactions but without DNA-binding domains, it is very likely that XAB2 was recruited to the promoter of CENPE by other proteins.

However, in the CENPE rescue experiment, we observed no significant restoration of cell cycle arrest when CENPE was re-expressed after XAB2 depletion (data not shown). Intriguingly, re-expression of CENPE after its own knockdown in Hela cells could not reverse cell cycle arrest either (data not shown). Possible explanation for these observations may include that the overexpression level is not high enough to compensate the depletion due to the high molecular weight of CENPE (312 kDa), or the phenotype induced by CENPE deficiency is severe and irreversible. In addition, we cannot exclude the possibility that the effect of XAB2 depletion is mediated by defects in multiple genes as revealed by microarray analysis that a subset of genes involving in cell cycle and mitotic progression are down-regulated.

Mitosis is one of the critical processes in cell cycle for proper chromosome segregation during cell division. Mitosis dysregulation often causes genome instability or aneuploidy, leads to mitotic catastrophe and cell death, and is closely associated with cancers and many other diseases. Thus, targeting mitosis has been proposed as an attractive therapeutic strategy for cancer therapy,^45, 46^ for example, CENPE inhibitor like GSK923295,^47^ syntelin^48^ or PF2721^49^ is now considered to have antitumour activity. Therefore, it will be interesting to investigate whether XAB2 can serve as a new anti-mitotic target for cancer therapy.

## Materials and Methods

### Constructs and antibodies

XAB2 construct was purchased from Origene and re-cloned into modified pcDNA3.1 vector (Promega, USA) containing HA tag at the 5′ end. A 1355 bp fragment of 5′ region sequence extending from −1263 to 92 (+1 is the transcription start site) of human CENPE gene was amplified by PCR from Hela genomic DNA and cloned into pGL3-Basic vector (Promega) at KpnI/HindIII sites. Deletion constructs of CENPE promoter were amplified from the full length promoter construct using nested PCR. The sequences of all the constructs were confirmed by direct sequencing. Primer sequences are listed in Supplementary Table 2.

Polyclonal antibody against XAB2 (Proteintech, Wuhan, China, 1 : 800), Phospho-Histone H3 (Ser10) (CST, MA, USA, 1:1000), Cdc2 (CST, 1:2000), Histone H2A.X (Proteintech, 1:1000), Phospho-Histone H2A.X (*γ*-H2A.X) (CST, 1:800), monoclonal antibodies against HA tag (Covance, MA, USA, 1:2000), CyclinB1 (CST, 1:2000), CENPE (Abcam, MA, USA, 1:1000), and *α*-tubulin (Sigma, Germany, 1:5000) were used in western blots.

### Cell culture and RNA interference

HeLa and 293T cells were gifts from Reed Lab in Harvard Medical School, MDA-MB-231 cells were purchased from the American Type Culture Collection (Manassas, VA, USA). All the cells were cultured in dulbecco's modified eagle medium supplemented with 10% fetal bovine serum.

For siRNA mediated gene knockdown, cells were plated in six-well plate and 2 *μ*l siRNA at 50 *μ*M were transfected using Lipofectamine 3000. Cells were treated with CENPE siRNA or XAB2 siRNA for analysis 24 or 55 h, respectively, after transfection. CENPE siRNA and XAB2 siRNA were previously reported,^22, 50^ non-target siRNA (RiboBio, China) was used as negative control. The siRNA sequences are as follows: CENPE siRNA (5′-CCACUAGAGUUGAAAGAUAdTdT-3′), XAB2-siRNA-1 (5′-CCAAUUCUCUGUCAAAUGCdTdT-3′), XAB2-siRNA-2 (5′-ACGCAGCACUCUCGAAUUUdTdT-3′).

For shRNA mediated gene knockdown, lentiviruses expressing XAB2 shRNA were produced as following: 293T cells were placed in 6 cm dish and cotransfected with 1.4 *μ*g of XAB2 shRNA (cloned in PLKO.1), 1.4 *μ*g of pREV, 1.4 *μ*g of pGag/Pol/PRE and 0.7 *μ*g of pVSVG. For control, replace XAB2 shRNA construct with control vector. Six hours after transfection, the mixture was replaced by fresh dulbecco's modified eagle medium containing 10% FBS and cells were cultured for an additional 48 h. Then the medium containing lentiviruses was filtered and stored at −80 °C. As for lentivirus infection, cells grown in six-well plate were infected twice with lentivirus containing 6 *μ*g/ml polybrene for a total of 48 h. For microarray, western blot, immunofluorescence, RT-PCR and cell cycle distribution analysis, cells were harvested after culture in fresh medium for another 24 h. For cell death analysis, cells were harvested 48 h later. The full sequence of XAB2 shRNA-1 and XAB2 shRNA-2 are CCGGGCTTCGCTACATCGAGTTCAACTCGAGTTGAACTCGATGTAGCGAAGCTTTTTG and CCGGGCAGTATGACATGTTCAACATCTCGAGATGTTGAACATGTCATACTGCTTTTTG, respectively. XAB2 shRNA-1 was used in the XAB2 knockdown experiments in this study unless otherwise indicated.

### Microarray gene expression analysis

Hela cells were plated in six-well plate and infected twice with control or XAB2 shRNA lentivirus. Then cells were harvested and total RNA was isolated using RNeasy plus kit (Qiagen, Germany) following the manufacturer's instruction. The integrity of total RNA was monitored with Agilent 2100 Bioanalyzer. Microarrays were performed using Human Transcriptome Array 2.0 (Affymetrix, USA) with three independent experiments. Results were analysed by using the Transcriptome Analysis Console from Affymetrix. Only genes that had a 2-fold increase or decrease in expression with a significance of *P*<0.05 were included in the final results. Functional analysis of the genes with expression significantly changed by XAB2 knockdown was performed using Gene Ontology analysis.

### FACS analysis

To examine the cell cycle distribution, cells were harvested and fixed in 75% ethanol at 4 °C overnight, washed twice with PBS, and then incubated with PI (Sigma) solution containing RNase for 30 min. The flow cytometry analysis was conducted on ACCURIC6, and the data were analysed using FlowJo7.6 software.

For cell death analysis, cells were stained using annexin/PI staining kit (KeyGEN BioTECH, Nanjing, China) for detection according to the manufacturer's instructions.

To detect the fraction of phospho-Histone H3 Ser10 positive cells, cells were fixed by 75% ethanol, permeabilized by 0.25% Triton X-100, and then stained with Alexa Fluor488-conjugated phospho-Histone H3 Ser10 antibody (1:50, CST) and PI.

### Immunofluorescence

Cells were grown in 35 mm cell culture dish with glass bottom (NEST, Wuxi, China), fixed with 4% paraformaldehyde (Sigma, Germany) for 30 min, followed by permeabilization with 0.5% Triton X-100 for 10 min at room temperature. Cells were then incubated with *α*-tubulin antibody (1:250) diluted in PBS with 10% calf serum at 4 °C overnight, and immunostained with Alexa 488-labeled anti-mouse antibody (1:500) diluted in the same buffer at room temperature for 30 min, followed by DAPI staining and four washes with PBS for 5 min each. Fluorescence was detected using DMI6000 microscope (Leica, Germany).

### Live cell imaging

GFP-H2B HeLa cells were a gift from Dr Huiyan Li in China National Center of Biomedical Analysis and were placed in 35 mm cell culture dish with glass bottom (NEST). Twenty-four hours after second infection with control or XAB2 shRNA lentivirus, images were captured every 10 min for 48 h using DMI6000 microscope (Leica) equipped with a × 40 objective lens in a chamber maintained at 37 °C with 5% CO_2_.

### Transfection and luciferase assay

Cells were seeded into 12-well plate the day before transfection. 1 μg of firefly luciferase constructs were transfected using lipofectamine 2000. The pRL-TK plasmid (100 ng/sample) encoding Renilla luciferase was cotransfected, and the readout was used for normalization of firefly luciferase activity. Cells were harvested 24 h after transfection and luciferase activity was measured with Dual-Luciferase Reporter Assay Kit (Promega). All transfections were performed in triplicate.

To assess the effect of XAB2 overexpression or knockdown on CENPE promoter activity, Hela cells in 12-well plates were transfected with XAB2 construct for 24 h or infected twice with lentivirus containing XAB2 shRNA, followed by co-transfection of CENPE promoter construct and pRL-TK vector, cells were then harvested after 24 h for the measurement of luciferase activity.

### ChIP

The ChIP assay was performed using EpiQuik Chromatin Immunoprecipitation Kit from Epigentek Group Inc. (Brooklyn, NY, USA) according to the manufacturer's protocol. Protein-DNA complexes were immunoprecipitated with HA antibody, and normal mouse IgG was used as negative control. Primer sets for CENPE were shown in Supplementary Table 2.

### Statistics

Data were presented as mean±s.d. (standard deviation). Statistical analyses between two groups were performed using Student's *t*-test with statistical significance defined as *P<0.05, **P<0.01 and ***P<0.001.

## Abbreviations

XPA: xeroderma pigmentosum group A
XAB2: XPA-binding protein 2
CSA: Cockayne syndrome group A
ATRA: all-trans retinoic acid
CENPE: centrosome-associated protein E
FACS: fluorescence-activated cell scanner
*γ*-H2A.X: Phospho-Histone H2A.X
ChIP: chromatin immunoprecipitation
s.d.: standard deviation
