# Supplementary figures and images for: XAB2 functions in mitotic cell cycle progression via transcriptional regulation of CENPE
Source: Cell Death Dis. 2016 Oct 13;7(10):e2409–. doi: 10.1038/cddis.2016.313 (PMC5133980; doi:10.1038/cddis.2016.313)

Fig. S1

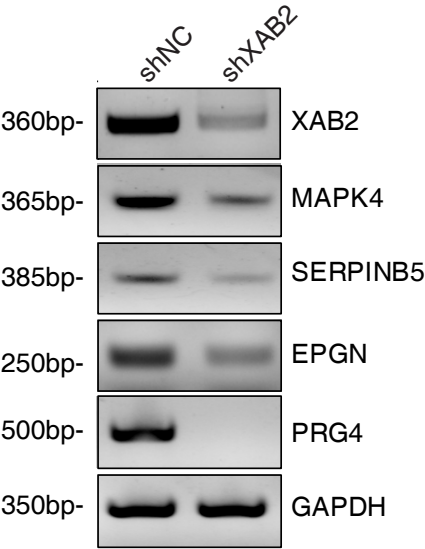

Supplement: Supplementary Figure S1 [file cddis2016313x1.pdf]

Fig. S2

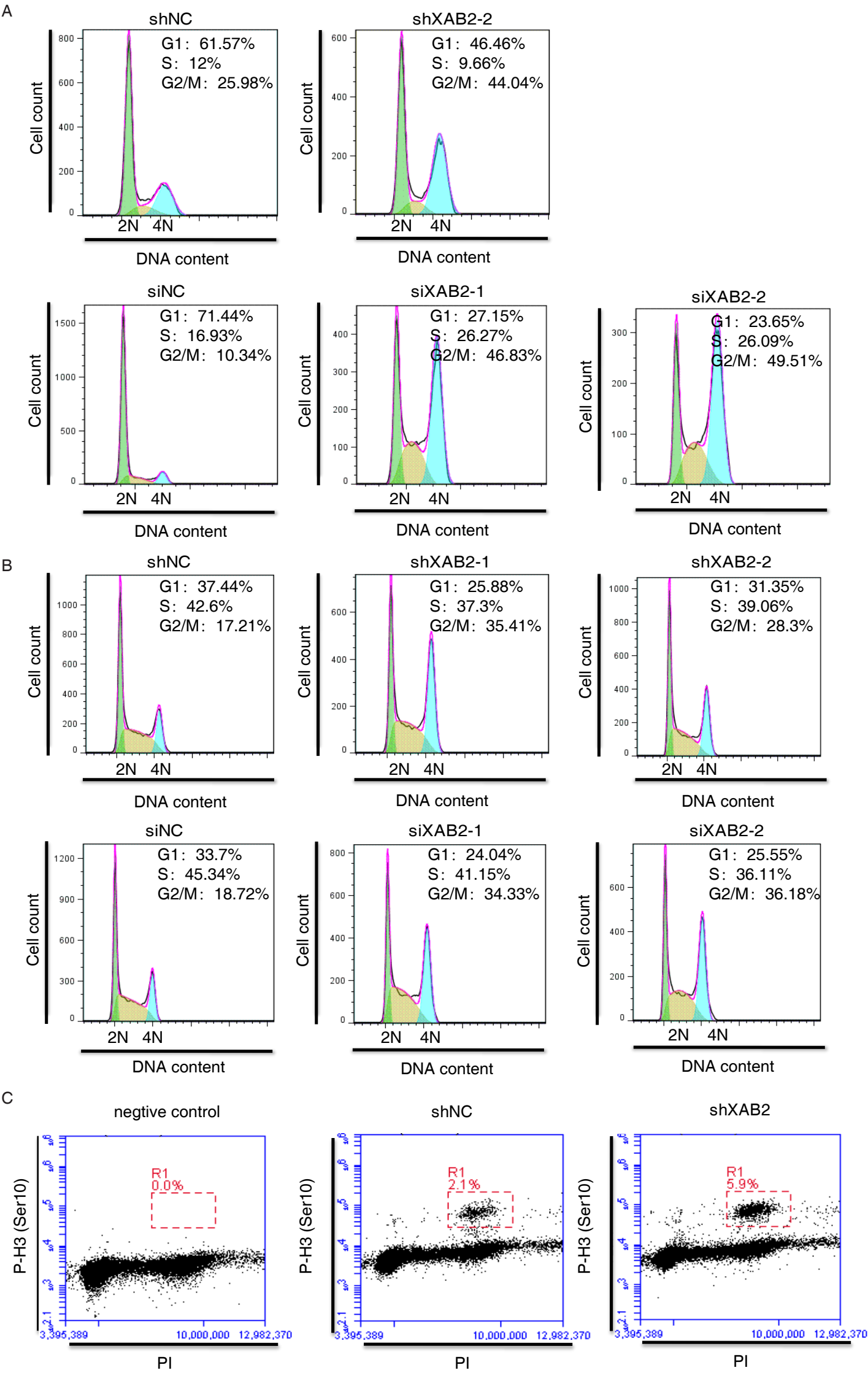

Supplement: Supplementary Figure S2 [file cddis2016313x2.pdf]

Fig. S3

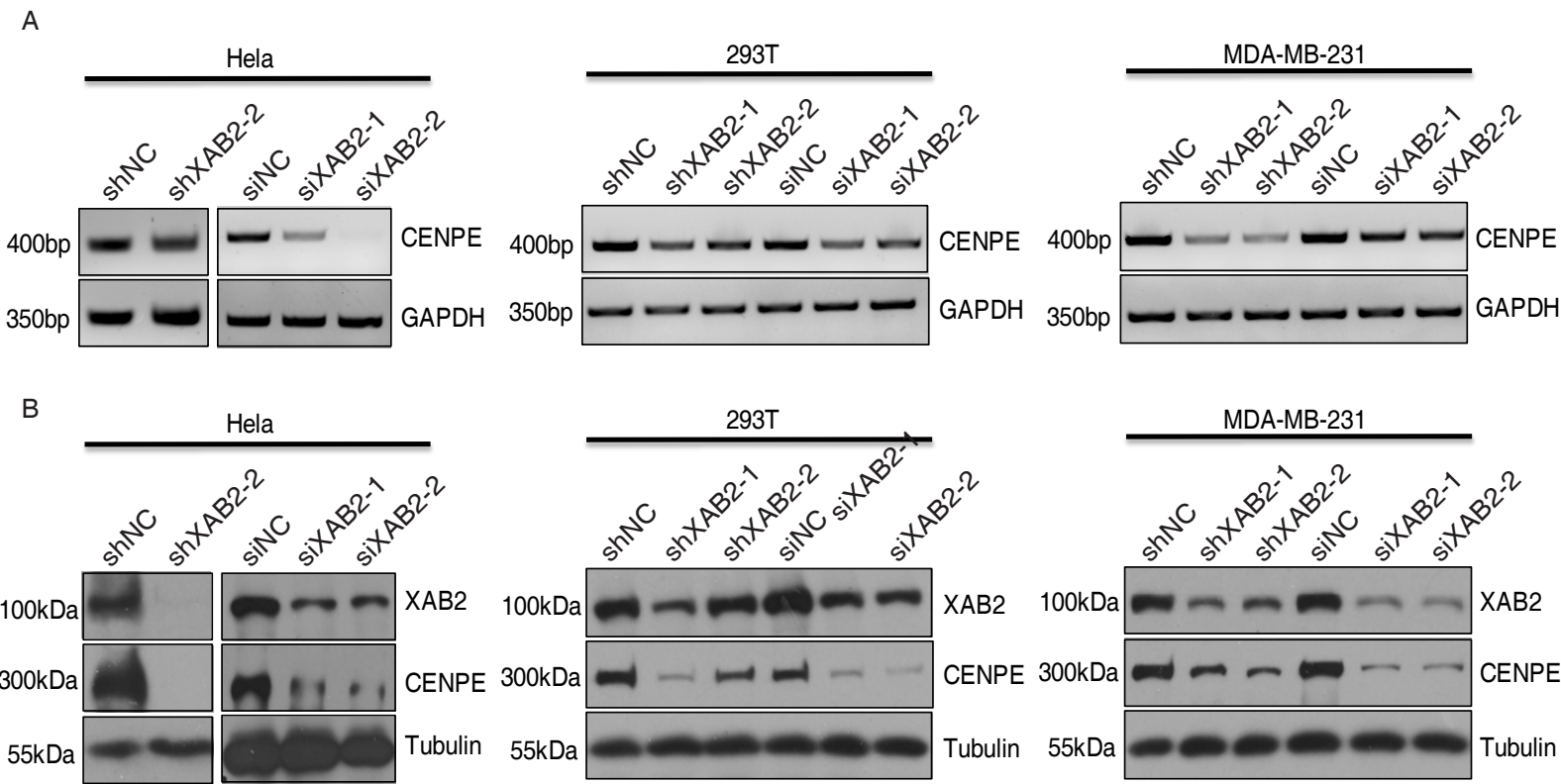

Supplement: Supplementary Figure S3 [file cddis2016313x3.pdf]
