# Supplementary material for: XAB2 functions in mitotic cell cycle progression via transcriptional regulation of CENPE
Source: Cell Death Dis. 2016 Oct 13;7(10):e2409–. doi: 10.1038/cddis.2016.313 (PMC5133980; doi:10.1038/cddis.2016.313)
Supplement: Supplementary Table S1 [file cddis2016313x4.pdf]

Table S1 Differential expression of genes (>2-fold, p<0.05) after XAB2 knockdown

Gene Symbol	Fold Change	Description
MIR4521	8.16	microRNA 4521
SNORD19	6.11	small nucleolar RNA, C/D box 19
CCL2	6.05	chemokine (C-C motif) ligand 2; NULL
SNX12	5.5	sorting nexin 12; NULL
SGK196; OTTHUMG00000164100	4.88	protein kinase-like protein SgK196; NULL
SNORD27; SNORD29; SNORD26; SNORD30; SNORD28; SNORD22; SNORD31; SNORD25; SNHG1	4.69	small nucleolar RNA, C/D box 27; small nucleolar RNA, C/D box 29; small nucleolar RNA, C/D box 26; small nucleolar RNA, C/D box 30; small nucleolar RNA, C/D box 28; small nucleolar RNA, C/D box 22; small nucleolar RNA, C/D box 31; small nucleolar RNA, C/D box 25; small nucleolar RNA host gene 1 (non-protein coding)
RPL30	4.57	NULL
PDRG1	4.24	p53 and DNA-damage regulated 1; NULL
TMEM109	4.05	transmembrane protein 109
HNRNPU-AS1	3.86	HNRNPU antisense RNA 1; NULL
RPIA; LOC101060545	3.81	ribose 5-phosphate isomerase A; ribose-5-phosphate isomerase-like; NULL
SFT2D2	3.78	SFT2 domain containing 2; NULL
SNORA32; SNORA1; SNORA18; SNORA8; MIR1304; SNORD5; TAF1D; SNORA40; SNORA25; SNORD6	3.75	small nucleolar RNA, H/ACA box 32; small nucleolar RNA, H/ACA box 1; small nucleolar RNA, H/ACA box 18; small nucleolar RNA, H/ACA box 8; microRNA 1304; small nucleolar RNA, C/D box 5; TATA box binding protein (TBP)-associated factor, RNA polymerase I, D, 41kDa; small nucleolar RNA, H/ACA box 40; small nucleolar RNA, H/ACA box 25; small nucleolar RNA, C/D box 6
MIR21	3.62	microRNA 21
HAUS2	3.6	HAUS augmin-like complex, subunit 2; NULL
NCBP2	3.56	nuclear cap binding protein subunit 2, 20kDa; NULL
TMEM30A	3.53	transmembrane protein 30A; NULL
AASDHPPT	3.53	aminoadipate-semialdehyde dehydrogenase-phosphopantetheinyl transferase
CDC42SE2	3.49	CDC42 small effector 2; NULL
ANKRD40	3.48	ankyrin repeat domain 40; NULL
SNORA5A	3.26	small nucleolar RNA, H/ACA box 5A

ERH	3.25	enhancer of rudimentary homolog (Drosophila)
RNU12	3.23	RNA, U12 small nuclear
ABHD17C	3.17	abhydrolase domain containing 17C
SNORD75	3.15	small nucleolar RNA, C/D box 75
SNHG17; OTTHUMG00000032442; RP4-564F22.2	3.12	small nucleolar RNA host gene 17 (non-protein coding); NULL
SNORA14B	3.11	small nucleolar RNA, H/ACA box 14B
ZNF830	3.11	zinc finger protein 830
TNFRSF10D	3.08	tumor necrosis factor receptor superfamily, member 10d, decoy with truncated death domain; NULL
PLEKHA3	3.05	pleckstrin homology domain containing, family A (phosphoinositide binding specific) member 3; NULL
SNORD69	3.01	small nucleolar RNA, C/D box 69
ODC1	3	ornithine decarboxylase 1; NULL
NR1D1	2.98	nuclear receptor subfamily 1, group D, member 1; NULL
STARD7	2.97	StAR-related lipid transfer (START) domain containing 7; NULL
ANP32B	2.97	acidic (leucine-rich) nuclear phosphoprotein 32 family, member B; NULL
VMA21	2.95	VMA21 vacuolar H+-ATPase homolog (S. cerevisiae); NULL
HIPK3	2.94	homeodomain interacting protein kinase 3;
LUZP6; MTPN	2.92	leucine zipper protein 6; myotrophin; NULL
YRDC	2.89	yrdC domain containing (E. coli); NULL
DESI2	2.88	desumoylating isopeptidase 2; NULL
SSX2B; SSX2	2.87	synovial sarcoma, X breakpoint 2B; synovial sarcoma, X breakpoint 2; NULL
SSX2B; SSX2	2.86	synovial sarcoma, X breakpoint 2B; synovial sarcoma, X breakpoint 2; NULL
SMN1; SMN2	2.85	survival of motor neuron 1, telomeric; survival of motor neuron 2, centromeric; NULL
RAB14	2.85	RAB14, member RAS oncogene family; NULL
SCARNA8	2.84	small Cajal body-specific RNA 8
CRKL	2.84	v-crk sarcoma virus CT10 oncogene homolog (avian)-like; NULL
NUS1	2.81	nuclear undecaprenyl pyrophosphate synthase 1 homolog (S. cerevisiae); NULL
C8orf33	2.81	chromosome 8 open reading frame 33

TAF4B	2.81	TAF4b RNA polymerase II, TATA box binding protein (TBP)-associated factor, 105kDa
SNORA13	2.8	small nucleolar RNA, H/ACA box 13
MIS12	2.79	MIS12, MIND kinetochore complex component, homolog (S. pombe); NULL
SDC4	2.79	syndecan 4; NULL
SPG21	2.78	spastic paraplegia 21 (autosomal recessive, Mast syndrome); NULL
SNORA65	2.77	small nucleolar RNA, H/ACA box 65
PTBP2	2.74	polypyrimidine tract binding protein 2; NULL
FNIP1; OTTHUMG00000162684; AC005593.1	2.74	folliculin interacting protein 1; NULL
SNORA9; SNHG15	2.73	small nucleolar RNA, H/ACA box 9; small nucleolar RNA host gene 15 (non-protein
TXNIP; LOC101060503	2.7	thioredoxin interacting protein; thioredoxin-interacting protein-like; NULL
CDC123	2.7	cell division cycle 123; NULL
PRPF39	2.7	PRP39 pre-mRNA processing factor 39 homolog (S. cerevisiae); NULL
RNA5SP108	2.68	RNA, 5S ribosomal pseudogene 108
NR1D2	2.68	nuclear receptor subfamily 1, group D, member 2; NULL
SMN1; SMN2	2.68	survival of motor neuron 1, telomeric; survival of motor neuron 2, centromeric; NULL
BRD2	2.68	NULL
TUSC2	2.67	tumor suppressor candidate 2; NULL
SNORA45	2.66	small nucleolar RNA, H/ACA box 45
SEC61B	2.65	Sec61 beta subunit; NULL
SNORD62B; SNORD62A	2.64	small nucleolar RNA, C/D box 62B; small nucleolar RNA, C/D box 62A
SNORD62B; SNORD62A	2.64	small nucleolar RNA, C/D box 62B; small nucleolar RNA, C/D box 62A
USP46	2.61	ubiquitin specific peptidase 46; NULL
GAN	2.61	gigaxonin; NULL
RNU5D-1	2.59	RNA, U5D small nuclear 1
UBE2G1	2.57	ubiquitin-conjugating enzyme E2G 1
GABPB1	2.56	GA binding protein transcription factor, beta subunit 1; NULL
SNORD51	2.55	small nucleolar RNA, C/D box 51
MIR3676	2.55	microRNA 3676
FYTTD1	2.53	forty-two-three domain containing 1; NULL
CBX1P1	2.53	chromobox homolog 1 pseudogene 1

SNORD4B	2.53	small nucleolar RNA, C/D box 4B
OVGP1	2.51	oviductal glycoprotein 1, 120kDa; NULL
API5	2.51	apoptosis inhibitor 5
CDCA5	2.51	cell division cycle associated 5
SNORA55	2.5	small nucleolar RNA, H/ACA box 55
YY1	2.47	YY1 transcription factor; NULL
SNORA5C	2.46	small nucleolar RNA, H/ACA box 5C
SNORD92	2.45	small nucleolar RNA, C/D box 92
MAD2L1	2.45	MAD2 mitotic arrest deficient-like 1 (yeast); NULL
TSPAN6	2.45	tetraspanin 6; NULL
BTBD10	2.45	BTB (POZ) domain containing 10
PMAIP1	2.45	phorbol-12-myristate-13-acetate-induced protein 1; NULL
SDE2	2.44	SDE2 telomere maintenance homolog (S. pombe); NULL
SNORD50A; SNHG5; SNORD50B	2.44	small nucleolar RNA, C/D box 50A; small nucleolar RNA host gene 5 (non-protein coding); small nucleolar RNA, C/D box 50B;
SNORD12C	2.44	small nucleolar RNA, C/D box 12C
SNORD85	2.43	small nucleolar RNA, C/D box 85
FOXJ3	2.42	forkhead box J3; NULL
SNORA53	2.4	small nucleolar RNA, H/ACA box 53
NHP2L1	2.4	NHP2 non-histone chromosome protein 2-like 1 (S. cerevisiae); NULL
SNORA10	2.39	small nucleolar RNA, H/ACA box 10
RNU6-30P	2.38	RNA, U6 small nuclear 30, pseudogene
NR2F2; MIR1469	2.38	nuclear receptor subfamily 2, group F, member 2; microRNA 1469; NULL
CBX1	2.38	chromobox homolog 1; NULL
TWISTNB	2.37	TWIST neighbor; NULL
RAD51AP1	2.37	RAD51 associated protein 1
CDKN2AIP	2.36	CDKN2A interacting protein; NULL
POLDIP3	2.36	polymerase (DNA-directed), delta interacting protein 3; NULL
RNU6-23P	2.34	RNA, U6 small nuclear 23, pseudogene
WDR45B; WDR45L	2.34	WD repeat domain 45B; WDR45-like; NULL
FBXO5	2.33	F-box protein 5; NULL
SNORD18B	2.33	small nucleolar RNA, C/D box 18B
HIVEP2	2.32	human immunodeficiency virus type I enhancer binding protein 2; NULL
OTUD3	2.31	OTU domain containing 3; NULL

RMND5A	2.31	required for meiotic nuclear division 5 homolog A (S. cerevisiae); NULL
RNF219	2.3	ring finger protein 219; NULL
SNORA38	2.3	small nucleolar RNA, H/ACA box 38
SNORA38	2.3	small nucleolar RNA, H/ACA box 38
SUB1	2.29	SUB1 homolog (S. cerevisiae); NULL
TMED9	2.29	transmembrane emp24 protein transport domain containing 9; NULL
SMNDC1	2.27	survival motor neuron domain containing 1;
USPL1	2.27	ubiquitin specific peptidase like 1; NULL
FAM103A1	2.27	family with sequence similarity 103, member A1; NULL
CKS1B	2.26	CDC28 protein kinase regulatory subunit 1B; NULL
ARL5A	2.26	ADP-ribosylation factor-like 5A; NULL
ACTL8	2.25	actin-like 8; NULL
SNORD101; SNORD100; SNORA33	2.25	small nucleolar RNA, C/D box 101; small nucleolar RNA, C/D box 100; small nucleolar RNA, H/ACA box 33
RCL1; OTTHUMG00000019476;	2.25	RNA terminal phosphate cyclase-like 1; NULL
WDR82	2.24	WD repeat domain 82; NULL
CREBZF	2.24	CREB/ATF bZIP transcription factor; NULL
RNU6-27	2.24	RNA, U6 small nuclear 27
MTFR2; FAM54A	2.23	mitochondrial fission regulator 2; NULL
SNORD11B	2.22	small nucleolar RNA, C/D box 11B
SNORA74A	2.22	small nucleolar RNA, H/ACA box 74A
SNORA28	2.22	small nucleolar RNA, H/ACA box 28
COPS7B	2.21	COP9 signalosome subunit 7B; COP9 constitutive photomorphogenic homolog subunit 7B (Arabidopsis); NULL
RNU6-80	2.21	RNA, U6 small nuclear 80
SNORA61; SNORA44; SNORA16A; SNHG12	2.2	small nucleolar RNA, H/ACA box 61; small nucleolar RNA, H/ACA box 44; small nucleolar RNA, H/ACA box 16A; small nucleolar RNA host gene 12 (non-protein coding); NULL
RNU6-45P	2.2	RNA, U6 small nuclear 45, pseudogene
PEBP1	2.2	phosphatidylethanolamine binding protein 1
PRPF3	2.19	pre-mRNA processing factor 3; NULL
RNU6-31P	2.19	RNA, U6 small nuclear 31, pseudogene
SNORA75	2.19	small nucleolar RNA, H/ACA box 75
SCML2	2.19	sex comb on midleg-like 2 (Drosophila); NULL

MAPRE1	2.19	microtubule-associated protein, RP/EB family, member 1; NULL
TMEM185B	2.18	transmembrane protein 185B
SMAD5	2.18	SMAD family member 5; NULL
RNU12-2P	2.18	RNA, U12 small nuclear 2, pseudogene
ALYREF	2.18	Aly/REF export factor; NULL
LRRC58	2.17	leucine rich repeat containing 58; NULL
SIAH2	2.17	siah E3 ubiquitin protein ligase 2; NULL
SNORA38	2.17	small nucleolar RNA, H/ACA box 38
RNU6ATAC	2.17	RNA, U6atac small nuclear (U12-dependent splicing)
WDR83OS	2.17	WD repeat domain 83 opposite strand
SNORA38	2.17	small nucleolar RNA, H/ACA box 38
SNORA38	2.17	small nucleolar RNA, H/ACA box 38
SNORA38	2.17	small nucleolar RNA, H/ACA box 38
SNORA38	2.17	small nucleolar RNA, H/ACA box 38
ZZZ3	2.16	zinc finger, ZZ-type containing 3; NULL
MED10	2.16	mediator complex subunit 10; NULL
C11orf58	2.16	chromosome 11 open reading frame 58
DDIT3	2.16	DNA-damage-inducible transcript 3
METTL21D	2.16	methyltransferase like 21D; NULL
SECISBP2L	2.16	SECIS binding protein 2-like
SNORD1A	2.16	small nucleolar RNA, C/D box 1A
SCARNA6	2.15	small Cajal body-specific RNA 6
ISY1	2.15	ISY1 splicing factor homolog (S. cerevisiae); NULL
MIR3143	2.15	microRNA 3143
SNORD11	2.14	small nucleolar RNA, C/D box 11
C16orf80	2.14	chromosome 16 open reading frame 80; NULL
PEX12	2.14	peroxisomal biogenesis factor 12; NULL
FBXO28	2.13	F-box protein 28; NULL
ATF1	2.13	activating transcription factor 1
TAF1A	2.12	TATA box binding protein (TBP)-associated factor, RNA polymerase I, A, 48kDa; NULL
HMGB2	2.12	high mobility group box 2; NULL
IER3	2.12	immediate early response 3; NULL
GLO1	2.12	glyoxalase I; NULL
RNU6-44	2.12	RNA, U6 small nuclear 44
BNIP2	2.12	BCL2/adenovirus E1B 19kDa interacting protein 2; NULL
TMEM33	2.11	transmembrane protein 33; NULL
SNORA84; MIR3651	2.11	small nucleolar RNA, H/ACA box 84; microRNA 3651

SSX3	2.11	synovial sarcoma, X breakpoint 3; NULL
SNORA76	2.11	small nucleolar RNA, H/ACA box 76
SNRPD1	2.11	small nuclear ribonucleoprotein D1 polypeptide 16kDa
KIAA0907	2.1	KIAA0907; NULL
EAF1	2.1	ELL associated factor 1; NULL
C10orf2	2.1	chromosome 10 open reading frame 2; NULL
BIRC5	2.1	baculoviral IAP repeat containing 5
ZNF557	2.1	zinc finger protein 557
RNU6-8	2.09	RNA, U6 small nuclear 8
SNHG8	2.09	small nucleolar RNA host gene 8 (non-protein coding)
ZFAND2A	2.09	zinc finger, AN1-type domain 2A; NULL
RBM18	2.09	RNA binding motif protein 18; NULL
BTBD7	2.08	BTB (POZ) domain containing 7
SNORA80	2.08	small nucleolar RNA, H/ACA box 80
NGDN	2.07	neuroguidin, EIF4E binding protein
NIPA2	2.07	non imprinted in Prader-Willi/Angelman syndrome 2; NULL
H3F3A; H3F3AP4; H3F3B	2.06	H3 histone, family 3A; H3 histone, family 3A, pseudogene 4; H3 histone, family 3B (H3.3B); NULL
RPS26; LOC100996747	2.06	ribosomal protein S26; 40S ribosomal protein S26-like
SNORD53	2.05	small nucleolar RNA, C/D box 53
CENPQ	2.05	centromere protein Q; NULL
MIR3153	2.05	microRNA 3153
RNU6-49	2.05	RNA, U6 small nuclear 49
SSX5	2.05	synovial sarcoma, X breakpoint 5; NULL
C1orf63	2.03	chromosome 1 open reading frame 63; NULL
SNORD70	2.03	small nucleolar RNA, C/D box 70
PRPF4B	2.03	PRP4 pre-mRNA processing factor 4 homolog B (yeast); NULL
MPZL3	2.03	myelin protein zero-like 3
CENPV	2.03	centromere protein V; NULL
SNORA68	2.03	small nucleolar RNA, H/ACA box 68
CRCP	2.02	CGRP receptor component; NULL
RNU6-38	2.02	RNA, U6 small nuclear 38
CYB5D1	2.02	cytochrome b5 domain containing 1
RNU6-4	2.01	RNA, U6 small nuclear 4
YY2	2.01	YY2 transcription factor; NULL
WEE1	2.01	WEE1 homolog (S. pombe)
DDX47	2.01	DEAD (Asp-Glu-Ala-Asp) box polypeptide 47

SNORA21	2.01	small nucleolar RNA, H/ACA box 21
RNU6-39P	2.01	RNA, U6 small nuclear 39, pseudogene
ARNT	-2.01	aryl hydrocarbon receptor nuclear translocator; NULL
SMARCAD1	-2.01	SWI/SNF-related, matrix-associated actin-dependent regulator of chromatin, subfamily a, containing DEAD/H box 1; NULL
KIAA1191	-2.01	KIAA1191; NULL
HERC4	-2.01	HECT and RLD domain containing E3 ubiquitin protein ligase 4; NULL
POLR3B	-2.01	polymerase (RNA) III (DNA directed) polypeptide B
SMARCC2	-2.01	SWI/SNF related, matrix associated, actin dependent regulator of chromatin, subfamily c, member 2
C18orf54	-2.01	chromosome 18 open reading frame 54; NULL
TNRC6B	-2.01	trinucleotide repeat containing 6B; NULL
LRPPRC	-2.02	leucine-rich pentatricopeptide repeat containing; NULL
EIF2AK3	-2.02	eukaryotic translation initiation factor 2-alpha kinase 3; NULL
PPARGC1A	-2.02	peroxisome proliferator-activated receptor gamma, coactivator 1 alpha; NULL
SPDL1; CCDC99	-2.02	spindle apparatus coiled-coil protein 1; coiled-coil domain containing 99; NULL
CCDC125	-2.02	coiled-coil domain containing 125; NULL
CGA	-2.02	glycoprotein hormones, alpha polypeptide
ZNF138	-2.02	zinc finger protein 138; NULL
KIAA0196	-2.02	KIAA0196; NULL
ZCCHC6	-2.02	zinc finger, CCHC domain containing 6; NULL
CHD4	-2.02	chromodomain helicase DNA binding protein 4
CSRP2	-2.02	cysteine and glycine-rich protein 2
EDC3	-2.02	enhancer of mRNA decapping 3; NULL
DOCK7	-2.03	dedicator of cytokinesis 7; NULL
CHML	-2.03	choroideremia-like (Rab escort protein 2);
KAT2B	-2.03	K(lysine) acetyltransferase 2B; NULL
HAUS6	-2.03	HAUS augmin-like complex, subunit 6; NULL
IDE	-2.03	insulin-degrading enzyme; NULL
CRY1	-2.03	cryptochrome 1 (photolyase-like)
COG6	-2.03	component of oligomeric golgi complex 6;
TRIP11	-2.03	thyroid hormone receptor interactor 11
ELP2	-2.03	elongator acetyltransferase complex subunit 2; NULL

SMC6	-2.04	structural maintenance of chromosomes 6;
OTTHUMG00000013250; RP11-187C18.2	-2.04	NULL
NAA35	-2.04	N(alpha)-acetyltransferase 35, NatC auxiliary subunit; NULL
TMEM133	-2.04	transmembrane protein 133
C11orf71	-2.04	chromosome 11 open reading frame 71
COQ10A	-2.04	coenzyme Q10 homolog A (S. cerevisiae)
VPS13C	-2.04	vacuolar protein sorting 13 homolog C (S. cerevisiae); NULL
PARN	-2.04	poly(A)-specific ribonuclease
TOM1L1	-2.04	target of myb1 (chicken)-like 1
KIAA1109	-2.05	KIAA1109; NULL
HSPA4L	-2.05	heat shock 70kDa protein 4-like; NULL
C5orf34	-2.05	chromosome 5 open reading frame 34; NULL
CENPK	-2.05	centromere protein K; NULL
IKBKAP	-2.05	inhibitor of kappa light polypeptide gene enhancer in B-cells, kinase complex-associated protein; NULL
CTPS2	-2.05	CTP synthase 2; NULL
TMEM135	-2.05	transmembrane protein 135
KIAA1377	-2.05	KIAA1377
USH1C	-2.05	Usher syndrome 1C (autosomal recessive,
RDX	-2.05	radixin
A2M	-2.05	alpha-2-macroglobulin; NULL
SUPT6H	-2.05	suppressor of Ty 6 homolog (S. cerevisiae)
MYH10	-2.05	myosin, heavy chain 10, non-muscle; NULL
MAVS	-2.05	mitochondrial antiviral signaling protein;
SEC22B; LOC100996517	-2.06	SEC22 vesicle trafficking protein homolog B (S. cerevisiae) (gene/pseudogene); vesicle-trafficking protein SEC22b-like
DISP1	-2.06	dispatched homolog 1 (Drosophila); NULL
SLC30A6	-2.06	solute carrier family 30 (zinc transporter), member 6; NULL
EPB41L5	-2.06	erythrocyte membrane protein band 4.1 like 5; NULL
LOC643401; OTTHUMG00000161968; RP11-46C20.1	-2.06	uncharacterized LOC643401; NULL
PRKDC	-2.06	protein kinase, DNA-activated, catalytic polypeptide; NULL
INVS	-2.06	inversin; NULL
FKTN	-2.06	fukutin; NULL

DCAF15	-2.06	DDB1 and CUL4 associated factor 15
AGL	-2.07	amylo-alpha-1, 6-glucosidase, 4-alpha-glucanotransferase; NULL
ASAP2	-2.07	ArfGAP with SH3 domain, ankyrin repeat and PH domain 2; NULL
LGR4	-2.07	leucine-rich repeat containing G protein-coupled receptor 4; NULL
USP2	-2.07	ubiquitin specific peptidase 2
DENND5B	-2.07	DENN/MADD domain containing 5B
FANCI	-2.07	Fanconi anemia, complementation group I;
GTF3C1	-2.07	general transcription factor IIIC, polypeptide 1, alpha 220kDa
DNMT1	-2.07	DNA (cytosine-5-)-methyltransferase 1
COX20	-2.08	COX20 cytochrome C oxidase assembly factor; NULL
LOC100506963; OTTHUMG00000002959; RP5-886K2.3	-2.08	uncharacterized LOC100506963; NULL
ZCCHC11	-2.08	zinc finger, CCHC domain containing 11;
ZNF512	-2.08	zinc finger protein 512; NULL
HLTF	-2.08	helicase-like transcription factor; NULL
OTTHUMG00000161756; RP11-269F21.3	-2.08	NULL
ERBB2IP	-2.08	erbb2 interacting protein; NULL
SNX9	-2.08	sorting nexin 9; NULL
KIF20B	-2.08	kinesin family member 20B; NULL
SCYL1	-2.08	SCY1-like 1 (S. cerevisiae)
RAB30	-2.08	RAB30, member RAS oncogene family
MPZL2	-2.08	myelin protein zero-like 2
GOLGA5	-2.08	golgin A5
IREB2	-2.08	iron-responsive element binding protein 2;
TNFAIP1	-2.08	tumor necrosis factor, alpha-induced protein 1 (endothelial); NULL
TMEM69	-2.09	transmembrane protein 69; NULL
TRAK2	-2.09	trafficking protein, kinesin binding 2; NULL
TFAP2A	-2.09	transcription factor AP-2 alpha (activating enhancer binding protein 2 alpha); NULL
RAET1E	-2.09	retinoic acid early transcript 1E; NULL
EMC2	-2.09	ER membrane protein complex subunit 2;
SECISBP2	-2.09	SECIS binding protein 2; NULL
CSTF3	-2.09	cleavage stimulation factor, 3' pre-RNA, subunit 3, 77kDa
AKAP13	-2.09	A kinase (PRKA) anchor protein 13

ACLY	-2.09	ATP citrate lyase; NULL
SPAG9	-2.09	sperm associated antigen 9; NULL
HCN2	-2.09	hyperpolarization activated cyclic nucleotide-gated potassium channel 2
SHKBP1	-2.09	SH3KBP1 binding protein 1
CDC45	-2.09	cell division cycle 45; NULL
INTS3	-2.1	integrator complex subunit 3; NULL
CPS1	-2.1	carbamoyl-phosphate synthase 1, mitochondrial; NULL
SORBS2	-2.1	sorbin and SH3 domain containing 2; NULL
VPS13A	-2.1	vacuolar protein sorting 13 homolog A (S. cerevisiae); NULL
C2CD5	-2.1	C2 calcium-dependent domain containing 5
NUP88	-2.1	nucleoporin 88kDa; NULL
RAD23A	-2.1	RAD23 homolog A (S. cerevisiae)
CTPS1	-2.11	CTP synthase 1; NULL
MAB21L3	-2.11	mab-21-like 3 (C. elegans); NULL
HES1	-2.11	hairy and enhancer of split 1, (Drosophila);
BMP2K	-2.11	BMP2 inducible kinase; NULL
KIF11	-2.11	kinesin family member 11; NULL
MIR548AN	-2.11	microRNA 548an
TARSL2	-2.11	threonyl-tRNA synthetase-like 2; NULL
ME2	-2.11	malic enzyme 2, NAD(+)-dependent, mitochondrial; NULL
LAMA1	-2.11	laminin, alpha 1; NULL
MN1	-2.11	meningioma (disrupted in balanced translocation) 1; NULL
HYAL1	-2.12	hyaluronoglucosaminidase 1; NULL
HUWE1	-2.12	HECT, UBA and WWE domain containing 1, E3 ubiquitin protein ligase; NULL
KIF18A	-2.12	kinesin family member 18A
PARP4	-2.12	poly (ADP-ribose) polymerase family, member 4; NULL
SCFD1	-2.12	sec1 family domain containing 1; NULL
ADAT1	-2.12	adenosine deaminase, tRNA-specific 1; NULL
CCDC18	-2.13	coiled-coil domain containing 18; NULL
SAP130	-2.13	Sin3A-associated protein, 130kDa; NULL
CDC23	-2.13	cell division cycle 23; NULL
BMS1	-2.13	BMS1 ribosome biogenesis factor; NULL
FAM45A; FAM45B	-2.13	family with sequence similarity 45, member A; family with sequence similarity 45, member A pseudogene; NULL
KIAA1731	-2.13	KIAA1731

EMP1	-2.13	epithelial membrane protein 1
MED29	-2.13	mediator complex subunit 29
OTTHUMG00000010753; RP4-714D9.2	-2.14	NULL
LRRC16A	-2.14	leucine rich repeat containing 16A; NULL
KLHDC3	-2.14	kelch domain containing 3; NULL
TGFBR1	-2.14	transforming growth factor, beta receptor 1;
NPY4R; LOC100996758; PPYR1	-2.14	neuropeptide Y receptor Y4; neuropeptide Y receptor type 4-like; NULL
DCLRE1A	-2.14	DNA cross-link repair 1A; NULL
RDH11	-2.14	retinol dehydrogenase 11 (all-trans/9-cis/11-cis)
TUBG1	-2.14	tubulin, gamma 1
CHAF1B	-2.14	chromatin assembly factor 1, subunit B (p60); NULL
ANKRD28	-2.15	ankyrin repeat domain 28; NULL
GOLIM4	-2.15	golgi integral membrane protein 4; NULL
NEK1	-2.15	NIMA-related kinase 1; NULL
LRRC1	-2.15	leucine rich repeat containing 1; NULL
TPP2	-2.15	tripeptidyl peptidase II; NULL
EPB41	-2.16	erythrocyte membrane protein band 4.1 (elliptocytosis 1, RH-linked); NULL
OTTHUMG00000161676; RP11-164P12.5	-2.16	NULL
RWDD4	-2.16	RWD domain containing 4; NULL
PJA2	-2.16	praja ring finger 2, E3 ubiquitin protein ligase; NULL
PGM3	-2.16	phosphoglucomutase 3; NULL
EZH2	-2.16	enhancer of zeste homolog 2 (Drosophila);
COQ9	-2.16	coenzyme Q9 homolog (S. cerevisiae)
SLC16A4	-2.17	solute carrier family 16, member 4 (monocarboxylic acid transporter 5); NULL
CLASP1	-2.17	cytoplasmic linker associated protein 1; NULL
CXorf61	-2.17	chromosome X open reading frame 61; NULL
FNDC3A	-2.17	fibronectin type III domain containing 3A;
RBL2	-2.17	retinoblastoma-like 2 (p130); NULL
MED11	-2.17	mediator complex subunit 11
SLFN5	-2.17	schlafen family member 5
VRK3	-2.17	vaccinia related kinase 3
OTTHUMG00000165064; RP11-255B23.4	-2.18	NULL
USP9X	-2.18	ubiquitin specific peptidase 9, X-linked; NULL
BBIP1	-2.18	BBSome interacting protein 1; NULL

PUS7L	-2.18	pseudouridylate synthase 7 homolog (S. cerevisiae)-like
LINC00052	-2.18	long intergenic non-protein coding RNA 52
TMEM189; TMEM189-UBE2V1; UBE2V1	-2.18	transmembrane protein 189; TMEM189-UBE2V1 readthrough; ubiquitin-conjugating enzyme E2 variant 1; NULL
CLCA2	-2.19	chloride channel accessory 2; NULL
MTOR	-2.19	mechanistic target of rapamycin (serine/threonine kinase); NULL
PLEKHG1	-2.19	pleckstrin homology domain containing, family G (with RhoGef domain) member 1; NULL
PRR11	-2.19	proline rich 11
COPA	-2.2	coatomer protein complex, subunit alpha;
TM4SF1-AS1	-2.2	TM4SF1 antisense RNA 1; NULL
OTTHUMG00000159595; RP11-278L15.2	-2.2	NULL
ANKRD32	-2.2	ankyrin repeat domain 32; NULL
LINC00473	-2.2	long intergenic non-protein coding RNA 473; NULL
PTER	-2.2	phosphotriesterase related; NULL
SLC35C2	-2.2	solute carrier family 35, member C2; NULL
INADL	-2.21	InaD-like (Drosophila); NULL
CLSPN	-2.21	claspin; NULL
YAE1D1	-2.21	Yae1 domain containing 1; NULL
SMIM2-AS1	-2.21	SMIM2 antisense RNA 1; NULL
LAMA3	-2.21	laminin, alpha 3; NULL
CASP14	-2.21	caspase 14, apoptosis-related cysteine peptidase
ARHGAP29	-2.22	Rho GTPase activating protein 29; NULL
RFESD	-2.22	Rieske (Fe-S) domain containing; NULL
OTTHUMG00000163343; CTB-43E15.3	-2.22	NULL
KRIT1; OTTHUMG00000155860; AC000120.7	-2.22	KRIT1, ankyrin repeat containing; NULL
ZMYM2	-2.22	zinc finger, MYM-type 2; NULL
TMC7	-2.22	transmembrane channel-like 7; NULL
WIPI1	-2.22	WD repeat domain, phosphoinositide
THOC1	-2.22	THO complex 1
IGF2R	-2.23	insulin-like growth factor 2 receptor; NULL
MYBL1	-2.23	v-myb myeloblastosis viral oncogene homolog (avian)-like 1; NULL
IDH3A	-2.23	isocitrate dehydrogenase 3 (NAD+) alpha;
TLR3	-2.24	toll-like receptor 3; NULL

PPP1R12A	-2.24	protein phosphatase 1, regulatory subunit 12A
HEATR1	-2.25	HEAT repeat containing 1; NULL
ANKRD36; ANKRD36C	-2.25	ankyrin repeat domain 36; ankyrin repeat domain 36C; NULL
SPINK13	-2.25	serine peptidase inhibitor, Kazal type 13 (putative); NULL
ISCA1P1	-2.25	iron-sulfur cluster assembly 1 homolog (S. cerevisiae) pseudogene 1
UBR5	-2.25	ubiquitin protein ligase E3 component n-recognin 5; NULL
CDKL5	-2.25	cyclin-dependent kinase-like 5; NULL
RTKN2	-2.25	rhotekin 2; NULL
PDE3B	-2.25	phosphodiesterase 3B, cGMP-inhibited
WNK1	-2.25	WNK lysine deficient protein kinase 1; NULL
CAMK1G	-2.26	calcium/calmodulin-dependent protein kinase IG; NULL
MYO6	-2.26	myosin VI; NULL
KIAA0368	-2.26	KIAA0368; NULL
METTL7A	-2.26	methyltransferase like 7A
UBA2	-2.26	ubiquitin-like modifier activating enzyme 2
UBE4B	-2.27	ubiquitination factor E4B; NULL
DRAM2	-2.27	DNA-damage regulated autophagy modulator 2; NULL
AGFG1	-2.27	ArfGAP with FG repeats 1; NULL
NMD3	-2.27	NMD3 homolog (S. cerevisiae); NULL
PDE8B	-2.27	phosphodiesterase 8B; NULL
C6orf203	-2.27	chromosome 6 open reading frame 203; NULL
UST	-2.27	uronyl-2-sulfotransferase; NULL
OTTHUMG00000164738; KB-1991G8.1	-2.27	NULL
ARFGEF1	-2.27	ADP-ribosylation factor guanine nucleotide-exchange factor 1 (brefeldin A-inhibited);
OTTHUMG00000019964; RP11-88I18.2	-2.27	NULL
NBEAL1; LOC648771	-2.28	neurobeachin-like 1; 60S ribosomal protein L12-like; NULL
GBF1	-2.28	golgi brefeldin A resistant guanine nucleotide exchange factor 1; NULL
SLC43A3	-2.28	solute carrier family 43, member 3
KDM5B	-2.29	lysine (K)-specific demethylase 5B; NULL
PSME4	-2.29	proteasome (prosome, macropain) activator subunit 4; NULL
WDR48	-2.29	WD repeat domain 48; NULL

CCNG1	-2.29	cyclin G1; NULL
ANKRD52	-2.29	ankyrin repeat domain 52
CDC42BPB	-2.29	CDC42 binding protein kinase beta (DMPK-
NF1	-2.29	neurofibromin 1; NULL
EML4	-2.3	echinoderm microtubule associated protein like 4; NULL
RICTOR	-2.3	RPTOR independent companion of MTOR, complex 2; NULL
OTTHUMG00000018428; RP11-472G21.2	-2.3	NULL
PNRC2; LOC100996626	-2.31	proline-rich nuclear receptor coactivator 2; proline-rich nuclear receptor coactivator 2-like;
DOCK10	-2.31	dedicator of cytokinesis 10; NULL
HMMR	-2.31	hyaluronan-mediated motility receptor (RHAMM); NULL
UTRN	-2.31	utrophin; NULL
STK38	-2.31	serine/threonine kinase 38; NULL
ZC2HC1A	-2.31	zinc finger, C2HC-type containing 1A; NULL
TLN1	-2.31	talin 1; NULL
DOCK11	-2.31	dedicator of cytokinesis 11; NULL
LOC92249; OTTHUMG00000021699; RP11-357C3.3	-2.31	uncharacterized LOC92249; NULL
PKP2	-2.31	plakophilin 2
SLC12A6	-2.31	solute carrier family 12 (potassium/chloride transporters), member 6; NULL
GFPT1	-2.32	glutamine--fructose-6-phosphate transaminase 1; NULL
ZNF860	-2.32	zinc finger protein 860; NULL
AHR	-2.32	aryl hydrocarbon receptor; NULL
VIPAS39	-2.32	VPS33B interacting protein, apical-basolateral polarity regulator, spe-39 homolog
OTTHUMG00000073673; RP11-126K1.8	-2.33	NULL
RHOB	-2.33	ras homolog family member B; NULL
MME	-2.33	membrane metallo-endopeptidase; NULL
OGDH	-2.33	oxoglutarate (alpha-ketoglutarate) dehydrogenase (lipoamide); NULL
ATAD2	-2.33	ATPase family, AAA domain containing 2;
INSL4	-2.33	insulin-like 4 (placenta); NULL
FBXO18	-2.33	F-box protein, helicase, 18; NULL
ADD3	-2.33	adducin 3 (gamma); NULL
ARHGAP12	-2.33	Rho GTPase activating protein 12; NULL

MCAM	-2.33	melanoma cell adhesion molecule
PPIP5K1	-2.33	diphosphoinositol pentakisphosphate kinase 1
METTL23	-2.33	methyltransferase like 23
AFF1	-2.34	AF4/FMR2 family, member 1; NULL
DAB2	-2.34	Dab, mitogen-responsive phosphoprotein, homolog 2 (Drosophila); NULL
DENND4C	-2.34	DENN/MADD domain containing 4C; NULL
CDC27	-2.34	cell division cycle 27
GPSM2	-2.35	G-protein signaling modulator 2; NULL
KIAA0922	-2.35	KIAA0922; NULL
TRAPPC11	-2.35	trafficking protein particle complex 11; NULL
PCSK1	-2.35	proprotein convertase subtilisin/kexin type 1; NULL
SLK	-2.35	STE20-like kinase; NULL
GOT1	-2.35	glutamic-oxaloacetic transaminase 1, soluble; glutamic-oxaloacetic transaminase 1, soluble (aspartate aminotransferase 1); NULL
MYO19	-2.35	myosin XIX
MOCOS	-2.35	molybdenum cofactor sulfurase; NULL
UBR4	-2.36	ubiquitin protein ligase E3 component n-recognin 4; NULL
ECT2	-2.36	epithelial cell transforming sequence 2 oncogene; NULL
LDB1	-2.36	LIM domain binding 1; NULL
ARNTL	-2.36	aryl hydrocarbon receptor nuclear translocator-like; NULL
CSE1L	-2.36	CSE1 chromosome segregation 1-like (yeast); NULL
HCP5	-2.36	HLA complex P5 (non-protein coding); NULL
PEX3	-2.37	peroxisomal biogenesis factor 3; NULL
NAV2	-2.37	neuron navigator 2; NULL
MRPL24	-2.38	mitochondrial ribosomal protein L24; NULL
OTTHUMG00000154381; AC064834.2	-2.38	NULL
LOC100996862; ANKRD36C	-2.38	ankyrin repeat domain-containing protein 36A-like; NULL
FCHO2	-2.38	FCH domain only 2; NULL
OTTHUMG00000014752; RP5-1120P11.1	-2.38	NULL
CHD9	-2.38	chromodomain helicase DNA binding protein 9
PITPNA	-2.38	phosphatidylinositol transfer protein, alpha
DPM1	-2.38	dolichyl-phosphate mannosyltransferase polypeptide 1, catalytic subunit; NULL

PHLDB2	-2.39	pleckstrin homology-like domain, family B, member 2; NULL
DST; LOC100652766	-2.39	dystonin; dystonin-like; NULL
PNRC2	-2.39	proline-rich nuclear receptor coactivator 2
KLRC3	-2.39	killer cell lectin-like receptor subfamily C, member 3
MPHOSPH9	-2.39	M-phase phosphoprotein 9
TMEM131	-2.4	transmembrane protein 131; NULL
ORC2	-2.4	origin recognition complex, subunit 2; NULL
DZIP3	-2.4	DAZ interacting zinc finger protein 3; DAZ interacting protein 3, zinc finger; NULL
SUPT20H; FAM48A	-2.4	suppressor of Ty 20 homolog (S. cerevisiae); family with sequence similarity 48, member A; NULL
MYO9A	-2.4	myosin IXA; NULL
DHPS	-2.4	deoxyhypusine synthase
ROBO1	-2.41	roundabout, axon guidance receptor, homolog 1 (Drosophila); NULL
MPDZ	-2.41	multiple PDZ domain protein; NULL
NUP160	-2.41	nucleoporin 160kDa
MMP12	-2.41	matrix metallopeptidase 12 (macrophage
AKTIP	-2.41	AKT interacting protein; NULL
DDX42	-2.41	DEAD (Asp-Glu-Ala-Asp) box helicase 42
PITPNC1	-2.41	phosphatidylinositol transfer protein,
AGPS	-2.42	alkylglycerone phosphate synthase; NULL
ALG1L13P	-2.42	asparagine-linked glycosylation 1-like 13, pseudogene
FSTL3	-2.42	follistatin-like 3 (secreted glycoprotein)
EEA1	-2.43	early endosome antigen 1
FAM208A	-2.44	family with sequence similarity 208, member A; NULL
UBA3	-2.44	ubiquitin-like modifier activating enzyme 3;
SPTAN1	-2.44	spectrin, alpha, non-erythrocytic 1; NULL
MYPN	-2.44	myopalladin; NULL
KIF21A	-2.44	kinesin family member 21A
SLC41A2	-2.44	solute carrier family 41, member 2; NULL
COQ5	-2.44	coenzyme Q5 homolog, methyltransferase (S. cerevisiae)
DENND4A	-2.44	DENN/MADD domain containing 4A
CTC1	-2.44	CTS telomere maintenance complex component
CDC42BPA	-2.45	CDC42 binding protein kinase alpha (DMPK-like); NULL
MYO1E	-2.45	myosin IE

DESI1	-2.45	desumoylating isopeptidase 1; NULL
FNBP1L	-2.46	formin binding protein 1-like; NULL
RHOQP2	-2.46	ras homolog family member Q pseudogene 2
DDX60	-2.46	DEAD (Asp-Glu-Ala-Asp) box polypeptide 60; NULL
RAD17	-2.46	RAD17 homolog (S. pombe); NULL
PPP3CB	-2.46	protein phosphatase 3, catalytic subunit, beta isozyme; NULL
TPR	-2.47	translocated promoter region, nuclear basket protein; NULL
PEX1	-2.47	peroxisomal biogenesis factor 1; NULL
MYBL2	-2.47	v-myb myeloblastosis viral oncogene homolog (avian)-like 2; NULL
POLA2	-2.48	polymerase (DNA directed), alpha 2, accessory subunit
TPCN1	-2.49	two pore segment channel 1
SNX1	-2.49	sorting nexin 1
KRCC1	-2.5	lysine-rich coiled-coil 1; NULL
TJP1	-2.5	tight junction protein 1; NULL
PITPNA-AS1	-2.5	PITPNA antisense RNA 1; NULL
ATP2B4	-2.51	ATPase, Ca++ transporting, plasma membrane 4; NULL
C10orf76	-2.51	chromosome 10 open reading frame 76; NULL
DYNC1H1	-2.51	dynein, cytoplasmic 1, heavy chain 1
TOP2A	-2.51	topoisomerase (DNA) II alpha 170kDa; NULL
AHCYL1	-2.52	adenosylhomocysteinase-like 1; NULL
SLC35A5	-2.52	solute carrier family 35, member A5; NULL
FER	-2.52	fer (fps/fes related) tyrosine kinase; NULL
PPFIBP1	-2.52	PTPRF interacting protein, binding protein 1 (liprin beta 1)
NRG4	-2.52	neuregulin 4; NULL
RSPRY1	-2.52	ring finger and SPRY domain containing 1;
ZCCHC17	-2.53	zinc finger, CCHC domain containing 17;
RAB3GAP2; AURKAPS1	-2.53	RAB3 GTPase activating protein subunit 2 (non-catalytic); aurora kinase A pseudogene 1;
LIPH	-2.53	lipase, member H; NULL
SAMD9	-2.54	sterile alpha motif domain containing 9; NULL
LINC00294	-2.54	long intergenic non-protein coding RNA 294
SASS6	-2.55	spindle assembly 6 homolog (C. elegans);
SMC4	-2.55	structural maintenance of chromosomes 4;
TRIP13	-2.55	thyroid hormone receptor interactor 13; NULL

PIK3C2A	-2.55	phosphatidylinositol-4-phosphate 3-kinase, catalytic subunit type 2 alpha; phosphoinositide-3-kinase, class 2, alpha
OIP5	-2.55	Opa interacting protein 5; NULL
AARS	-2.55	alanyl-tRNA synthetase
ZNF831	-2.55	zinc finger protein 831; NULL
CITED4	-2.56	Cbp/p300-interacting transactivator, with Glu/Asp-rich carboxy-terminal domain, 4;
EIF2AK4	-2.56	eukaryotic translation initiation factor 2 alpha kinase 4
RNFT1	-2.56	ring finger protein, transmembrane 1; NULL
KYNU	-2.57	kynureninase; NULL
TCAIM	-2.57	T cell activation inhibitor, mitochondrial;
CLIP1	-2.57	CAP-GLY domain containing linker protein 1
ITPR1	-2.58	inositol 1,4,5-trisphosphate receptor, type 1; NULL
C14orf162; CCDC177	-2.58	chromosome 14 open reading frame 162; coiled-coil domain containing 177
RAB11B-AS1	-2.58	RAB11B antisense RNA 1
RBBP9	-2.58	retinoblastoma binding protein 9; NULL
RAP2A	-2.59	RAP2A, member of RAS oncogene family;
U2SURP	-2.6	U2 snRNP-associated SURP domain containing; NULL
MIR548W	-2.61	microRNA 548w
HPS3	-2.62	Hermansky-Pudlak syndrome 3; NULL
ITGA2	-2.62	integrin, alpha 2 (CD49B, alpha 2 subunit of VLA-2 receptor); NULL
ATP2B1	-2.62	ATPase, Ca++ transporting, plasma membrane
LMO7	-2.62	LIM domain 7; NULL
ATP7A	-2.64	ATPase, Cu++ transporting, alpha polypeptide; NULL
TMEM259	-2.64	transmembrane protein 259
ABCC2	-2.65	ATP-binding cassette, sub-family C (CFTR/MRP), member 2; NULL
MYLK3	-2.65	myosin light chain kinase 3; NULL
OTTHUMG00000159786; RP11-85F14.5	-2.66	NULL
KLHDC2	-2.66	kelch domain containing 2; NULL
NCAPD3	-2.67	non-SMC condensin II complex, subunit D3
FAM83B	-2.68	family with sequence similarity 83, member B; NULL
WDHD1	-2.68	WD repeat and HMG-box DNA binding protein 1; NULL

OTTHUMG00000163317; RP11-195E2.4	-2.7	NULL
NF1P5	-2.71	neurofibromin 1 pseudogene 5
CRYBG3	-2.73	beta-gamma crystallin domain containing 3
OTTHUMG00000132913; AC012146.7	-2.73	NULL
STAM2	-2.75	signal transducing adaptor molecule (SH3 domain and ITAM motif) 2; NULL
CENPI	-2.75	centromere protein I; NULL
FAM5C	-2.76	family with sequence similarity 5, member C; NULL
CCDC88A	-2.76	coiled-coil domain containing 88A; NULL
JAGN1	-2.76	jagunal homolog 1 (Drosophila); NULL
TAF11	-2.76	TAF11 RNA polymerase II, TATA box binding protein (TBP)-associated factor, 28kDa; NULL
UFL1	-2.78	UFM1-specific ligase 1; NULL
LINC00958	-2.78	long intergenic non-protein coding RNA 958
KNTC1	-2.79	kinetochore associated 1
SH3PXD2B	-2.8	SH3 and PX domains 2B; NULL
ARHGAP21	-2.81	Rho GTPase activating protein 21; NULL
CKAP5	-2.81	cytoskeleton associated protein 5
TRANK1	-2.82	tetratricopeptide repeat and ankyrin repeat containing 1; NULL
DLGAP1-AS1	-2.82	DLGAP1 antisense RNA 1
IBTK	-2.83	inhibitor of Bruton agammaglobulinemia tyrosine kinase; NULL
ZWILCH	-2.83	zwilch kinetochore protein; NULL
NAE1	-2.84	NEDD8 activating enzyme E1 subunit 1; NULL
FLVCR2	-2.85	feline leukemia virus subgroup C cellular receptor family, member 2
BRIP1	-2.87	BRCA1 interacting protein C-terminal helicase
NDRG3	-2.87	NDRG family member 3; NULL
LOC100505876; OTTHUMG00000152162; AC007390.5	-2.89	uncharacterized LOC100505876; NULL
FAM114A1	-2.89	family with sequence similarity 114, member A1; NULL
ZDHHC16	-2.89	zinc finger, DHHC-type containing 16; NULL
ADAL	-2.89	adenosine deaminase-like
OTTHUMG00000163424; CTC-295J13.3	-2.91	NULL
PDK4	-2.91	pyruvate dehydrogenase kinase, isozyme 4;
USO1	-2.92	USO1 vesicle transport factor; NULL

CCAT1; OTTHUMG00000165065; RP11-255B23.3	-2.93	colon cancer associated transcript 1 (non-protein coding); NULL
ITGA5	-2.93	integrin, alpha 5 (fibronectin receptor, alpha polypeptide)
ENOX2	-2.94	ecto-NOX disulfide-thiol exchanger 2; NULL
OTTHUMG00000154423; AC009948.5	-2.95	NULL
LIMCH1	-2.95	LIM and calponin homology domains 1; NULL
APPBP2	-2.96	amyloid beta precursor protein (cytoplasmic tail) binding protein 2
PPP2R3A	-2.97	protein phosphatase 2, regulatory subunit B", alpha; NULL
DPH5	-2.98	diphthamide biosynthesis 5; DPH5 homolog (S. cerevisiae); NULL
WDR44	-2.98	WD repeat domain 44; NULL
UBR1	-2.99	ubiquitin protein ligase E3 component n-recognin 1; NULL
HERC5	-3.02	HECT and RLD domain containing E3 ubiquitin protein ligase 5; NULL
FASN	-3.02	fatty acid synthase
ROCK1	-3.02	Rho-associated, coiled-coil containing protein kinase 1; NULL
CRYBG3	-3.03	beta-gamma crystallin domain containing 3;
MOSPD2	-3.04	motile sperm domain containing 2; NULL
SERPINB4	-3.07	serpin peptidase inhibitor, clade B (ovalbumin), member 4; NULL
DENND6A	-3.08	DENN/MADD domain containing 6A; NULL
UBR2	-3.09	ubiquitin protein ligase E3 component n-recognin 2; NULL
DOCK5	-3.09	dedicator of cytokinesis 5; NULL
NPC1	-3.12	Niemann-Pick disease, type C1; NULL
HEATR5A	-3.13	HEAT repeat containing 5A
PRIM1	-3.15	primase, DNA, polypeptide 1 (49kDa)
SPTLC3	-3.16	serine palmitoyltransferase, long chain base subunit 3; NULL
C8orf22	-3.18	chromosome 8 open reading frame 22; NULL
TRMT1L	-3.19	tRNA methyltransferase 1 homolog (S. cerevisiae)-like; NULL
RHOQ	-3.19	ras homolog family member Q; NULL
DHX32	-3.21	DEAH (Asp-Glu-Ala-His) box polypeptide 32; NULL

CTR9	-3.22	Ctr9, Paf1/RNA polymerase II complex component, homolog (S. cerevisiae)
MYO1C	-3.22	myosin IC; NULL
DNAJC13	-3.23	DnaJ (Hsp40) homolog, subfamily C, member 13; NULL
LXN	-3.23	latexin; NULL
ZNF252P	-3.24	zinc finger protein 252, pseudogene
UBA6	-3.26	ubiquitin-like modifier activating enzyme 6;
MKNK2	-3.32	MAP kinase interacting serine/threonine kinase
IDH1	-3.33	isocitrate dehydrogenase 1 (NADP+), soluble; NULL
SERPINB3	-3.36	serpin peptidase inhibitor, clade B (ovalbumin), member 3; NULL
CTH	-3.39	cystathionase (cystathionine gamma-lyase);
TRMT61B	-3.39	tRNA methyltransferase 61 homolog B (S. cerevisiae); NULL
TLR6	-3.4	toll-like receptor 6; NULL
KIAA0825	-3.4	KIAA0825; NULL
C15orf65	-3.43	chromosome 15 open reading frame 65
XAB2	-3.43	XPA binding protein 2
POLR2A	-3.55	polymerase (RNA) II (DNA directed) polypeptide A, 220kDa
CENPE	-3.56	centromere protein E, 312kDa; NULL
BRWD3	-3.58	bromodomain and WD repeat domain containing 3; NULL
KIAA1033	-3.59	KIAA1033
OTTHUMG00000015949; RP1-276N6.2	-3.61	NULL
MAPK4	-3.67	mitogen-activated protein kinase 4
PPME1	-3.68	protein phosphatase methylesterase 1
POP1	-3.75	processing of precursor 1, ribonuclease P/MRP subunit (S. cerevisiae); NULL
SERPINB5	-3.78	serpin peptidase inhibitor, clade B (ovalbumin), member 5; NULL
ROCK2	-3.82	Rho-associated, coiled-coil containing protein kinase 2; NULL
MCF2	-3.99	MCF.2 cell line derived transforming sequence; NULL
TLCD1	-4.01	TLC domain containing 1; NULL
DNAJC12	-4.02	DnaJ (Hsp40) homolog, subfamily C, member 12; NULL
STEAP4	-4.07	STEAP family member 4; NULL
PALMD; MIR548AA1	-4.15	palmdelphin; microRNA 548aa-1; NULL

OTTHUMG00000074333; AJ006998.2	-4.15	NULL
LOC100506498	-4.99	uncharacterized LOC100506498
EPGN	-5.87	epithelial mitogen homolog (mouse); epithelial mitogen; NULL
RAB31	-6	RAB31, member RAS oncogene family
PRG4	-9.03	proteoglycan 4; NULL
