# Supplementary material for: XAB2 functions in mitotic cell cycle progression via transcriptional regulation of CENPE
Source: Cell Death Dis. 2016 Oct 13;7(10):e2409–. doi: 10.1038/cddis.2016.313 (PMC5133980; doi:10.1038/cddis.2016.313)
Supplement: Supplementary Table S2 [file cddis2016313x5.pdf]

Table S2 Primers for cloning in this study

Cloning primer	Sequence(5'-3')
CENPE-p(-1263~92)-F	GATTGCTTGAGCCTAGGAGTTC
CENPE-p(-1263~92)-R	ATCCTATCAGGCTGAACTGGTC
CENPE-p(-1263~92)-KF	TATGGTACCGATTGCTTGAGCCTAGGAGTTC
CENPE-p(-1263~92)-HR	GAGAAGCTTATCCTATCAGGCTGAACTGGTC
CENPE-p(-808~92)-KF	TGAGGTACCAGGACTTTGAGACCAGCCTT
CENPE-p(-408~92)-KF	TATGGTACCTGGACTGGACAGCGAATTTC
CENPE-p(-208~92)-KF	TATGGTACCGTATTCCAGGACCACCACTC
CENPE-p(-58~92)-KF	TAAGGTACCGAATGGCCCATGACGTCACG
CENPE-p(-1263~-209)-HR	GCGAAGCTTCAGAATACTATTATCTGCCA
CENPE-p(-1263~-59)-HR	ATTAAGCTTCACGTGCGGAGCAGCGCGGG
CENPE-p(-408~-209)-ChIP-F	GACTGGACAGCGAATTTCAA
CENPE-p(-408~-209)-ChIP-R	AGCTAGCGAACGTCATCCTC
CENPE-p(-208~-59)-ChIP-F	TTCCAGGACCACCACTCTCT
CENPE-p(-208~-59)-ChIP-R	GTGACGTCATGGGCCATTC
XAB2-HindIII-F	TATAAGCTTGTGGTGATGGCGCGACTCTCGCGGCCCGA
XAB2-XbaI-R	AGGTCTAGATCAGTCTTCCTTCAGGCTCCCAAACACTGCGGCT
CENPE-RT-F	CCTTAACTTGTGGAGGTGGC
CENPE-RT-R	AGCGAACTGGATGAGGTGAT
CKAP5-RT-F	GAGAAAAGAGGCCCTGGAGT
CKAP5-RT-R	CCAACATGACATTGGTGTCC
CLIP1-RT-F	CTCTTCCCTCAGCTCCATGA
CLIP1-RT-R	ATGTGCTGCTGCTTCTCCTT
CDC27-RT-F	GTTCCCAAAGAATCCCTCGT
CDC27-RT-R	GTGTGTCATCCGCATCTGTC
MAD2L1-RT-F	TGGCCGAGTTCTTCTCATTC
MAD2L1-RT-R	TGGCAGAAATGTCACCGTAG
GAPDH-RT-F	CTGAGTACGTCGTGGAGTCC
GAPDH-RT-R	AGGCAGGGATGATGTTCTGG
